# Community-driven research and capacity building to address environmental justice concerns with industrial air pollution in Curtis Bay, South Baltimore

**DOI:** 10.3389/fepid.2023.1198321

**Published:** 2023-09-12

**Authors:** Matthew A. Aubourg, Greg Sawtell, Lauren Deanes, Nicole Fabricant, Meleny Thomas, Kristoffer Spicer, Caila Wagar, Shashawnda Campbell, Abigail Ulman, Christopher D. Heaney

**Affiliations:** ^1^ Community Science and Innovation for Environmental Justice (CSI EJ) Initiative, Center for a Livable Future, Department of Environmental Health and Engineering, Johns Hopkins Bloomberg School of Public Health, Baltimore, MD, United States; ^2^ Community of Curtis Bay Association, Curtis Bay, Baltimore, MD, United States; ^3^ South Baltimore Community Land Trust, Curtis Bay, Baltimore, MD, United States; ^4^ Department of Anthropology, Sociology, and Criminal Justice, Towson University, Towson, MD, United States; ^5^ Department of Epidemiology, Johns Hopkins Bloomberg School of Public Health, Baltimore, MD, United States; ^6^ Department of International Health, Johns Hopkins Bloomberg School of Public Health, Baltimore, MD, United States

**Keywords:** community-driven research, capacity building, environmental justice (EJ), environmental racism, hyperlocal air monitoring, industrial air pollution, community-academic partnerships, Baltimore

## Abstract

**Introduction:**

Curtis Bay (CB) is an environmental justice (EJ) community in South Baltimore. With a high concentration of industrial polluters and compounding non-chemical stressors, CB has experienced socioeconomic, quality of life, and health burdens for over 100 years. Today, these polluters include the open-air CSX Coal Terminal, waste-to-energy incinerators, and heavy diesel traffic through residential areas. The Community of Curtis Bay Association, Free Your Voice, and South Baltimore Community Land Trust are local organizations enacting a vision for equitable, healthy, and community-led development without industrial encroachment. In response to community-identified EJ concerns and an explosion at the CSX Coal Terminal, CB community groups partnered with academic researchers to develop a community-driven hyperlocal air monitoring and capacity building approach. This paper describes this approach to characterizing hyperlocal air quality in CB, building bridges between community residents and regulatory agencies, and nurturing a cohesive and effective community-academic partnership toward EJ.

**Methods:**

Using hyperlocal air monitoring, we are collecting real-time air pollution (particulate matter, black carbon, and ground-level gas species) and meteorological data from 15 low-cost sensors in residential and industrial areas of CB. We also use trail cameras to record activities at the CSX Coal Terminal. We merge air pollution and industrial activity data to evaluate the following: overall air quality in CB, multi-air pollutant profiles of elevated events, spatiotemporal changes in air quality in the community, patterns of industrial activity, and potential correlations between air quality and observed industrial activity. Members of our partnership also lead a high school course educating students about the history and ongoing efforts of the EJ movement in their community. Students in this course learn how to employ qualitative and quantitative data collection and analysis methods to bring scientific support to community EJ concerns.

**Results and Discussion:**

Our hyperlocal air monitoring network and community-academic partnership are continuing to evolve and have already demonstrated the ability to respond to community-identified EJ issues with real-time data while developing future EJ leaders. Our reflections can assist other community and academic groups in developing strong and fruitful partnerships to address similar EJ issues.

## 1. Introduction

Dr. Robert Bullard coined the term “human sacrifice zone” to describe communities presently inundated by environmental justice (EJ) issues and polluting industries while continuing to “attract new polluters” (1). Environmental justice is the indelible right to equitable environmental protection under the law; the ability to live safe, healthy, and productive lives; and possessing the decision-making power to effect positive environmental change. Communities afflicted by environmental injustice are often called EJ, fenceline, or frontline communities. “Frontline” connotes and invokes imagery of a battlefield. Yet, these battlegrounds are homes, community centers, schools, and playgrounds, predominantly in communities of color and lower socioeconomic status (SES). Similarly to a battlefield, however, the causalities and impacts upon quality of life are very real.

Fenceline communities are directly adjacent to or near industrial facilities and mobile pollution sources, placing residents at higher magnitude and duration of exposure to toxic pollution (i.e., air, water, and soil contamination) (2, 3). Polluting sources include diesel truck traffic, oil and natural gas extraction, waste management facilities, concentrated animal feeding operations, chemical manufacturers, and many other industries. Some emitted pollutants are shared between multiple source types (e.g., particulate matter (4, 5)) and others are unique to certain polluters or industries (e.g., hydrogen sulfide (6) and volatile organic compounds (7)). Ambient air pollution in fenceline communities is of particular concern to community members, researchers, and regulators. Air pollutants can invade nearby households, cause short- and long-term health impacts, and can reduce air quality far from their source.

Increased exposure to and the effects of industrial air pollution disproportionately impact communities of color and lower SES in the United States (US) (8–10). Historically inequitable and racist zoning, permitting, and siting practices have placed industrial polluting sources in vulnerable communities and driven people of color and/or low SES into industrialized areas (11, 12). Emissions from industrial polluters are often un- or under-regulated by local and state regulatory bodies (3, 13) which places environmental and health burdens upon communities structurally and historically dis-empowered in decision-making. Fires, toxic leaks, explosions, and other emergencies at industrial facilities also present health and safety hazards to fenceline residents (14, 15). As a result, already vulnerable communities are suffocated by the cumulative impacts of these environmental hazards and compounding non-chemical stressors (e.g., limited healthcare access, food insecurity, financial instability, and mental and psychosocial stressors) (16, 17).

The US environmental justice movement stems from the bravery and activism of community members in impacted neighborhoods, but has leveraged the power of scientific research to inform data-driven decision-making (18, 19). Fenceline and community air monitoring collect air quality data to address air pollution in EJ communities. Air monitoring technology has continued to develop with the proliferation of low-cost sensing technologies. Low-cost sensors (LCSs) are affordable, easy to deploy, and require low energy inputs while still providing high quality spatiotemporal air quality data (20, 21). Low-cost sensors have helped usher air monitoring out of its highly technical silo and into the hands of fenceline residents as tools bringing truth to power (22–24). Community science and community-engaged research (CEnR) as participatory approaches to air monitoring have evolved in parallel to LCS availability (25, 26).

Community-engaged research approaches center the knowledge and intrinsic power within a community and its residents (27). Community stakeholders possess understanding and lived experience that can shape research methods at all stages of a study. To acknowledge and combat a history of exploitative and transactional relationships between academic institutions and marginalized communities (28, 29), CEnR emphasizes co-learning amongst all team members and maintains community participation in the research process and dissemination (30, 31). Community science and CEnR in fenceline communities can generate robust locally and culturally relevant data for EJ efforts.

The community of Curtis Bay (CB) in South Baltimore, Maryland is a quintessential example of a human sacrifice zone and has been the chosen site of polluting industries for over 100 years. Curtis Bay community members have expressed concerns about industrial air pollution and its effect upon individual and community health for decades. Local industries, including multiple waste incinerators, coal-fired power plants, and heavy diesel traffic, are thoroughly documented in scientific literature as drivers of poor air quality (32–36).

Curtis Bay residents have long observed and identified the impact of air pollution in their daily lives. Strong, eye-watering odors, an uncanny feeling of “thick air,” and dust-blackened clothes while line-drying outdoors are some examples of these observations. Residents discussed keeping their windows closed for over a decade living in the community to prevent coal dust from entering their homes. Community members also note several friends, family members, and neighbors who have fallen ill or passed away from cancer and respiratory issues—health outcomes known to be related to industrial air pollution. Corroborating community lived experience, age-adjusted mortality rates in the CB area due to heart disease, lung cancer, chronic lower respiratory disease, and cancer of all kinds exceed complimentary rates in the rest of Baltimore City (Table 1) (37).

**Table 1:** Age-adjusted mortality rates due to heart disease, lung cancer, chronic lower respiratory disease, and cancer of all kinds in the Curtis Bay area (includes Curtis Bay, Brooklyn, and Hawkins Point neighborhoods) compared to the rest of Baltimore City.

	Age-adjusted mortality rate (Deaths per 10,000)	
	Curtis Bay Area	Baltimore City
Heart Disease	36.1	24.4
Lung Cancer	8.3	5.9
Chronic Lower Respiratory Disease	8.4	3.6
Cancer (all kinds)	24.2	21.2

On December 30, 2021, there was an explosion at the CSX Curtis Bay Coal Terminal. This facility is less than 1,000 feet from the community recreation center and nearby residences (38). The explosion shattered the windows of nearby homes. In response to the explosion, the Community of Curtis Bay Association (CCBA) and the South Baltimore Community Land Trust (SBCLT) requested community-engaged technical support from the Community Science and Innovation for Environmental Justice (CSI EJ) Initiative at Johns Hopkins Bloomberg School of Public Health and the University of Maryland Center for Community Engagement, Environmental Justice, and Health.

We designed a hyperlocal (limited to the local Curtis Bay geographical area) air monitoring network to bring scientific, data-driven, and responsive support to longstanding community concerns about industrial air pollution. Partners from Towson University, the CSI EJ research team, and SBCLT led a class at Curtis Bay's Benjamin Franklin High School (BFHS) to bring students into this monitoring project and the local EJ movement. Community leadership and participation is embedded at every stage in this air monitoring, youth empowerment, and capacity building approach to addressing EJ concerns in CB. This paper describes our community-driven approach to characterizing hyperlocal air quality in CB and developing effective partnerships to incite regulatory action.

## 2. Methods

### 2.1. History of environmental injustice in the Curtis Bay area

The threat of environmental injustice in the CB area can be traced back to a critical expansion or acceleration of rail in 1882 with the establishment of the Baltimore and Ohio Railroad coal pier (39)—known today as the CSX Curtis Bay Coal Terminal. Baltimore and Ohio Railroad sold CSX the open-air coal pier in the 1950s. The CSX Coal Terminal along with the nearby CNX Marine Terminal handled a combined 13.8 million short tons of coal in 2010 (40). Today, the CSX Coal Terminal alone has the capacity to move 14 million tons of coal annually (41). The expansion of coal has impacted the overall quality of life and environment of CB.

Two case examples of industrial overburden eventually displacing residents are the communities of Wagner's Point—a historically White community originally dedicated in the 1920s to canning—and Fairfield—a historically Black community where residents mainly worked on the Fairfield shipyards during the WWII era (42). Wagner's Point and Old Fairfield were dubbed the “industrial peninsula” as chemical and fertilizer companies joined wartime industry to encroach upon the communities. During this period of industrialization, land was bought by corporations and by Baltimore City, displacing residents, predominantly of color and low SES (42). Residents were offered minor remuneration as property value was low due to surrounding industrial zoning and related safety concerns. Residents of color who remained in the community were the “last hired, first fired” or never employed at all by the developing industrial facilities (42). Fairfield Homes, a public housing development, was predominantly White until the first Black family arrived in 1953. A few years later, the housing and broader community was nearly 100 percent Black. Residents in the Fairfield community did not receive basic sanitation services or plumbing until 1976. These two communities suddenly became engulfed by industries in the 1980s and 1990s and eventually the city of Baltimore offered a small monetary compensation for the last residents of Fairfield (42, 43). Here, we can see the ways in which industry encroaches upon community, social support systems, and eventually displaces residents.

Wartime industry was gradually replaced by expansion of waste industries in the industrial peninsula, coupled by City-wide divestment. The 1940s to 1990s are marked by this transition to waste-to-energy industries. Notably, the Baltimore Refuse Energy System Company's and the Curtis Bay Medical Waste Services' incinerators were established in 1983 and 1991, respectively. The Baltimore Refuse Energy System Company Incinerator alone can burn 2,250 tons of waste per day and consumes around 700,000 tons of waste per year (44). Garbage and medical waste incineration emissions—including particulate matter (PM), toxic dioxins, heavy metals, and greenhouse gases—are significant threats to human health and exacerbate climate change and ecological degradation (45–47). This expansion of waste-to-energy industries intensified concerns regarding respiratory health of the community and quality of life.

In 2011, a group of students from BFHS in CB learned about plans to build the nation's largest waste-to-energy incinerator a mile away from their school, marketed as a positive, renewable energy initiative (48). The proposed site for the incinerator was the same land where past Fairfield and Wagner's Point communities once resided prior to their displacement by industry. The students were already meeting after school to better understand the social and environmental conditions in their neighborhoods. After hearing about the incinerator and researching the associated negative community health impacts, they delved into Curtis Bay's history as an industrial “sacrifice zone.” The students called their group Free Your Voice (FYV) (43, 49). Seeing blatant connections between past injustice and current realities, they worked to successfully organize a political campaign called “Stop the Incinerator.” After canvassing the community, getting public entities to divest from buying cheap energy, and pressuring the Maryland Department of the Environment (MDE) to pull the facility's permits, they eventually halted the construction of the incinerator.

The courageous efforts of FYV led to the establishment of SBCLT. South Baltimore Community Land Trust is a 501(c)(3) focused on housing justice and zero waste which uses resident- and worker-led tools to advance community-defined development. Alongside CCBA, SBCLT developed a vision for CB community development centered on equitable community ownership of land and a just transition to zero waste in defiance of the prevailing waste-to-energy industry (50). The continued, overwhelming industrial presence in CB was of significant concern with years of documented nuisance complaints, high rates of poor health outcomes, and emergent events placing the health and safety of the community at risk. Other South Baltimore communities have been impacted by the same industry-related concerns within and outside of CB. Free Your Voice, SBCLT, and CCBA have built cross-community collaboration as part of the SB7 Coalition to form a concerted effort toward community empowerment and development (51).

### 2.2. Site description: Curtis Bay area today

**Figure 1:**
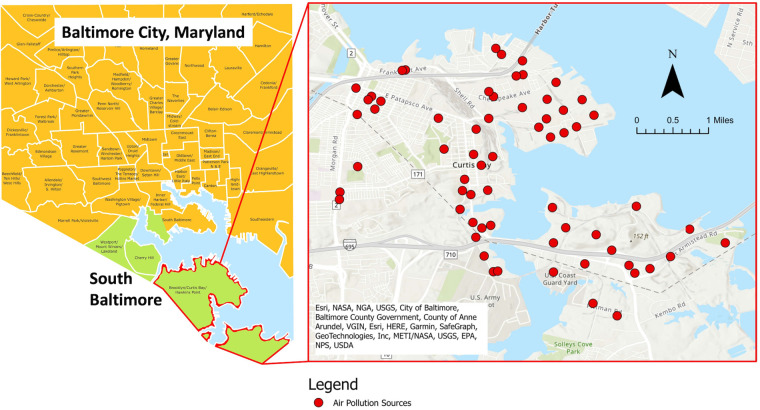
Clustering of Maryland Department of the Environment-regulated stationary sources of air pollution (red dots) in the Curtis Bay area.

As of 2017, the population size of the CB area (Brooklyn/Curtis Bay/Hawkins Point community statistical area) is 14,626 people (37). The median household income is $32,598 with 32.1% of households living below the poverty line (52). 68.3% of residents in the CB area are people of color, primarily Black/African American (35.8%). There are 1,013.2 vacant lots per 10,000 housing units which exceeds that of Baltimore City by 335.9 lots per 10,000 units (37). There is a significant industrial presence in the community with 80.9% of land area zoned for industrial use (37). There are around 70 MDE-regulated stationary sources of air pollution in CB (Figure 1), which emit a suite of harmful air pollutants, including the US Environmental Protection Agency's criteria air pollutants, volatile organic compounds, methane, and airborne heavy metals (53, 54). According to the US Environmental Protection Agency EJScreen and compared to all US census tracts, CB is in the 99th percentile for proximity to Risk Management Program sites; 96th percentile for proximity to hazardous waste facilities; 93rd percentile for wastewater discharges; 90th percentile for proximity to Superfund/National Priority List sites; and 80th to 90th percentiles for ozone exposure, diesel particulate matter, air toxics cancer risk, and air toxics respiratory hazard index (55).

In 2007 and 2008, 20.6 and 21.6 million pounds, respectively, of toxic air pollutants were emitted by polluters in CB—the greatest quantity of toxic air pollutants from stationary sources in the US (56). After improved pollution control technology was implemented at nearby coal—fired power plants, the magnitude of toxic pollutions dropped to 2.2 million pounds. Still, the CB zip code had the greatest magnitude of air pollution in Maryland state.

### 2.3. Developing research objectives

Our community-academic team compiled observations and community issues by attending community meetings and discussing long-standing concerns. Prolonged and on-the-ground activities in CB exposed academic team members to community EJ concerns firsthand such as industrial malodors and constant diesel truck traffic in residential areas. Together, the community-academic team developed primary research objectives based on community-identified issues alongside research capabilities of the academic partners.

#### 2.3.1. Short-term objectives

• Measure levels of air pollution in CB in terms of the following pollutants:
  ⚬ Particulate matter [PM_1_, PM_2.5_, PM_10_, and total suspended particles (TSP or PM_40_)]
  ⚬ Carbon monoxide (CO)
  ⚬ Carbon dioxide (CO_2_)
  ⚬ Nitric oxide (NO)
  ⚬ Nitrogen dioxide (NO_2_)
  ⚬ Ground-level ozone (O_3_)
  ⚬ Black carbon (BC)
  ⚬ Volatile organic compounds (VOCs)
• Determine the frequency, magnitude, and duration of elevated air pollution events in CB
• Estimate cumulative and source-specific impacts of industrial facilities on air quality in CB, including CB residents' concerns with the CSX Coal Terminal
• Use ground-truthing methods combining air pollutant concentration measurements and visual confirmation of industrial activity patterns for:
  ⚬ Air pollution source identification
  ⚬ Real-time health and safety alerts for community residents during emergent events
  ⚬ Data-driven enhancements to enforcement and compliance practices for ten industrial facilities seeking permit renewal in the next 3 years
• Develop a model and training materials for youth engagement in the local EJ movement with advocacy, leadership, and scientific skills building
• Cultivate multi-directional educational opportunities and co-learning between CB residents, researchers, students, and environmental regulators

#### 2.3.2. Mid- to long-term objectives

• Characterize cumulative impacts of stationary and mobile air pollution in CB
• Develop a sustainable, community-operated hyperlocal air monitoring network in CB
  ⚬ Create and implement training activities and education materials
  ⚬ Build infrastructure and organization for network longevity
• Utilize air monitoring data generated to inform and strengthen industrial permitting practices, bringing about structural permit reform and improving long-term community health
• Expand air monitoring network into other South Baltimore communities with shared and unique environmental health and justice concerns

Toward these objectives, we developed a community-driven hyperlocal network to monitor overall air quality and flag events of elevated air pollution. Community members can inform our team about observed environmental hazards such as chemical spills or dust plumes to isolate air quality data during and near the event. As the monitor maintenance and data analysis process is refined amongst our team, community members and local students will be trained to operate the network, analyze data, and develop visualizations such as air quality time series. Thereby, the hyperlocal air monitoring network will become a community-owned and -operated tool for pursuing EJ and improving community health.

### 2.4. Academic role in facilitating regulatory agency partnerships

Discussions with community residents revealed sentiments of mistrust in local and state environmental regulatory agencies. In 2008, MDE removed the only regulatory air monitor from near the CB area. When pressed by community residents to justify this decision, MDE did not provide a response. Some mistrust also stems from the perceived slow response and lack of communication following the explosion at the CSX Coal Terminal. Additionally, the long-term persistence of stationary and mobile industrial polluters in the community with seemingly little oversight influences community skepticism.

Leading up to our partnership, MDE acknowledged their failure to adequately staff and resource EJ issues in CB and South Baltimore. The MDE Air and Radiation Administration provides technical support in developing and maintaining the monitoring network, as well as data cleaning, quality control, and analysis. Regular meetings with the Air and Radiation Administration open a direct line of communication and cooperation between CB community representatives and regulatory stakeholders. Community partners give input in all parts of discussion to ensure decision-making power and transparency.

From the community perspective, the collaboration with MDE provides timely information and a space to interpret data and synthesize findings. This contributes to a greater level of empowerment within the CB community, particularly related to pushing MDE to set stronger standards and address longstanding EJ concerns. At the same time, the collaboration highlights the extent of the gaps in MDE's approach to regulating polluting industries in CB—in other words, there is still much work to be done.

We receive additional and essential technical support from the manufacturers of the air monitoring technology used in this initiative (QuantAQ, Inc. and Distributed Sensing Technologies, LLC). Representatives from the manufacturers join regular meetings and have visited the hyperlocal air monitoring network to provide on-the-ground technical support.

### 2.5. Hyperlocal air monitoring network

The Maryland Department of the Environment currently operates a regional ambient air monitoring network across Maryland state comprised of stations recording meteorological conditions, concentrations of ground-level criteria air pollutants, and air toxics (57). The nearest regulatory air monitor to CB is over 10 miles away and can characterize regional air quality and conditions, rather than community-level. The concentration distance decay of some pollutants of concern in the community (e.g., PM_10_ and black carbon) occurs in the order of tens to hundreds of meters (58). Therefore, a hyperlocal air monitoring network is required to measure community-level air quality instead of regional conditions measured by the MDE network. The hyperlocal network detects temporally shorter and geographically localized elevated air pollution events. The first node of the network was deployed in May 2022.

#### 2.5.1. Air quality monitoring with low-cost sensors in Curtis Bay

Multiple air monitor types were selected for this network to widen the set of pollutant parameters monitored while addressing pollutants of community concern (Table 2).

**Table 2:** Air quality monitors used and air pollutants/parameters measured in the community-driven hyperlocal air monitoring network.

Monitor	Parameters Measured	Manufacturer
MODULAIR with co-located sonic anemometer	• Particulate matter (PM_1_, PM_2.5_, PM_10_) • Total suspended particles (TSP or PM_40_) • Carbon monoxide (CO) • Carbon dioxide (CO_2_) • Nitric oxide (NO) • Nitrogen dioxide (NO_2_) • Ozone (O_3_) • Temperature • Relative humidity • Wind speed • Wind direction	QuantAQ, Inc.; Somerville, MA
ObservAir	• Particulate matter (PM_1_, PM_2.5_, PM_4_, PM_10_) • Black carbon (BC) • Carbon monoxide (CO) • Nitrogen dioxide (NO_2_) • Temperature • Relative humidity	Distributed Sensing Technologies, LLC; Richmond, CA

**Figure 2:**
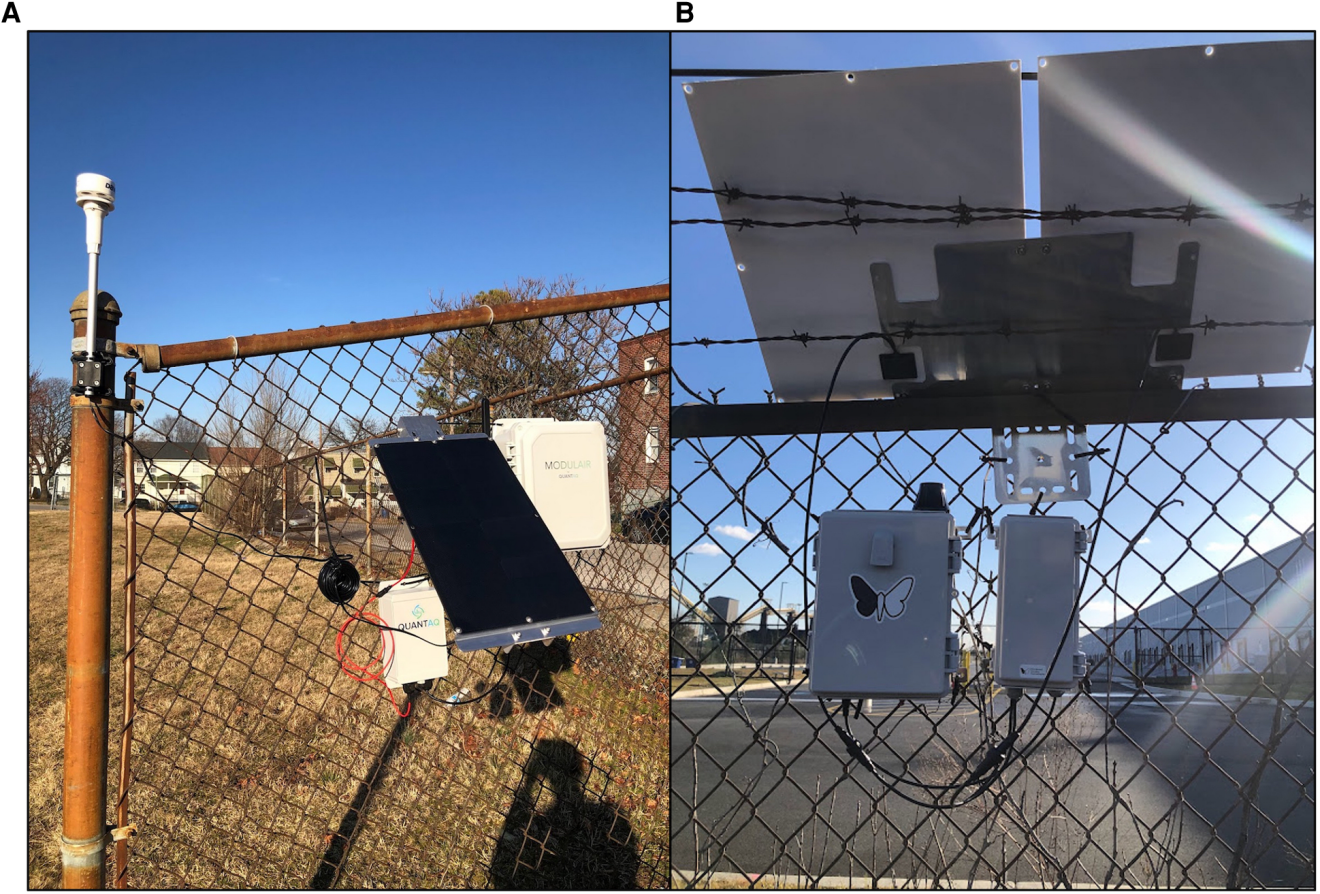
QuantAQ MODULAIR (QuantAQ, Inc.) (**A**) and Distributed Sensing Technologies ObservAir (Distributed Sensing Technologies, LLC) (**B**) air quality monitors are used in the community-driven hyperlocal air monitoring network.

QuantAQ MODULAIR (QuantAQ, Inc., Somerville, MA) sensors (*n* = 10) comprise the majority of instruments in the hyperlocal network (Figure 2A). Using an on-board optical particle counter and nephelometer, the MODULAIR provides real-time measurements of PM (PM_1_, PM_2.5_, and PM_10_) with 24 particle size bins ranging from 0.35 to 40.0 µm allowing for measurement of TSP. The MODULAIR also measures ground-level gas species—namely CO, CO_2_, NO, NO_2_, and O_3_—and environmental conditions (temperature and relative humidity). Each MODULAIR is connected to a sonic anemometer (Davis Instruments Corp., Hayward, CA) to record wind speed and direction. Each parameter measurement is uploaded via LTE to the QuantAQ Cloud at 1-minute resolution and is accessible to all members of the community-academic team.

We also use Distributed Sensing Technologies (DSTech) ObservAir monitors (Distributed Sensing Technologies, LLC, Richmond, CA) (*n* = 3) to measure concentrations of BC, PM_1_, PM_2.5_, PM_4_, PM_10_, gases (CO and NO_2_), and environmental conditions (temperature and relative humidity) (Figure 2B). Measurements are uploaded via LTE at 1-minute resolution and data is accessible to all members of the community-academic team. ObservAir monitors are co-located with MODULAIRs to correlate wind speed and direction data.

**Figure 3:**
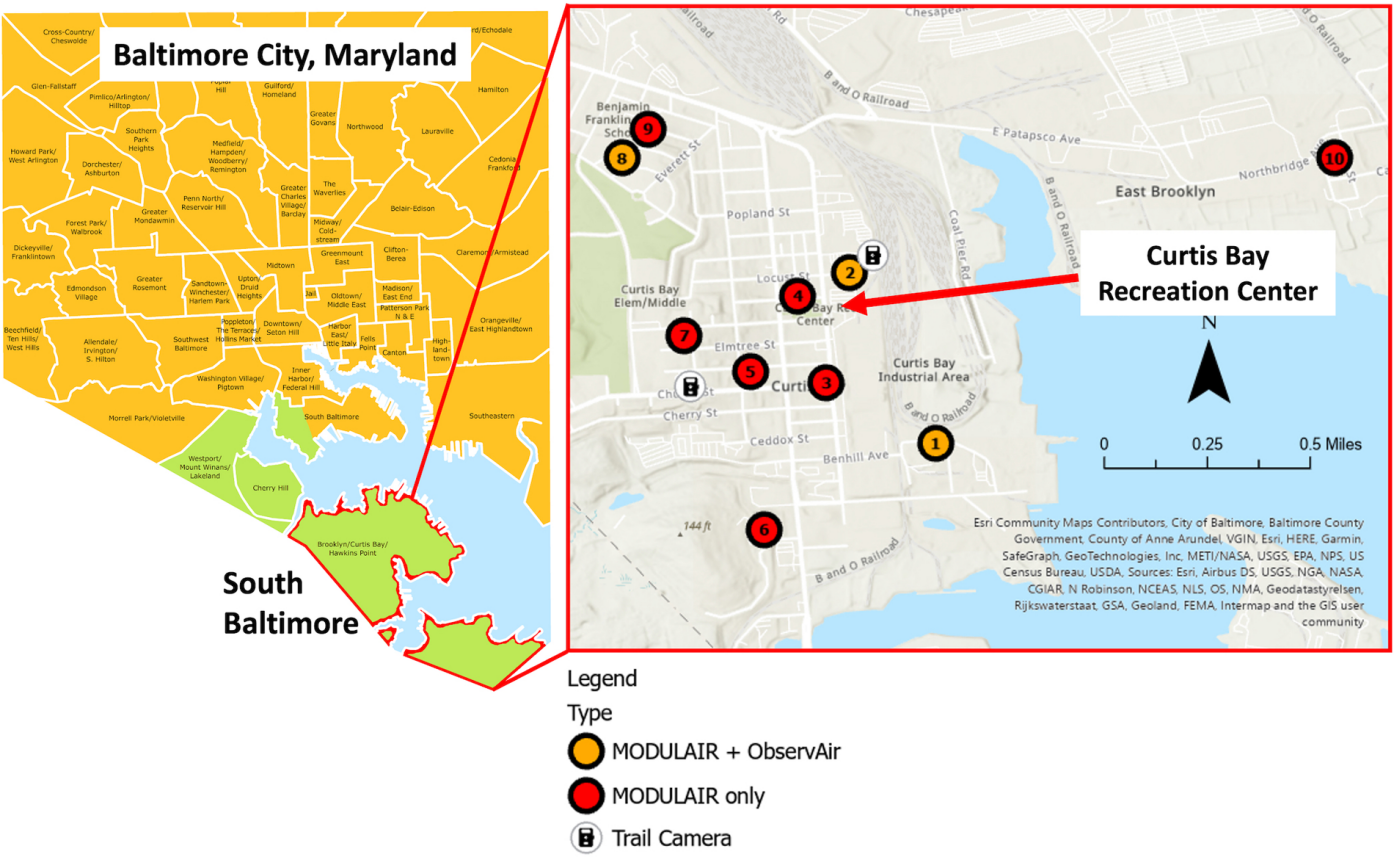
Community-driven hyperlocal air monitoring network in Curtis Bay, South Baltimore, Maryland. Monitoring sites with co-located MODULAIR and ObservAir monitors shown in orange. Monitoring sites with only MODULAIR monitors deployed shown in red. Locations of the two trail cameras are displayed with the camera icon. Locations are numbered from nearest to farthest distance from the CSX Curtis Bay Coal Terminal—an industrial polluter of primary community concern.

Air monitors are distributed around the CB community in residential and industrial areas (Figure 3). All monitors in the network are powered using solar panels except for one residential site directly connected to power due to deployment location constraints. Hosts (community residents and local businesses) and monitoring sites are identified and contacted by the community partners. We inform agreeing hosts about the project, obtain their oral consent, and give a cash incentive for their participation and to offset any associated costs. Monitoring sites are selected by their proximity to polluters of community concern, gap filling for air dispersion modelling, and feasibility of deployment. We use an iterative process for determining monitoring locations to meet the needs of hosts or improve deployment conditions.

Academic team members conduct weekly inspections and maintenance while responding to technical issues affecting data completeness (i.e., inadequate cellular connectivity, power outages due to lack of solar exposure, and other malfunctions). In some instances, community residents hosting monitors provide information about monitor status from visual inspection and conduct guided troubleshooting themselves.

#### 2.5.2. Ground-truthing with low-cost cameras and community lived experience

The community-driven hyperlocal network of air monitors is a component of our ground-truthing method and strategy. Ground-truthing methods combine *in situ* visual observations with collected measurements. This contributes further data for source identification and improved communication of findings to lay and technical stakeholders.

**Figure 4:**
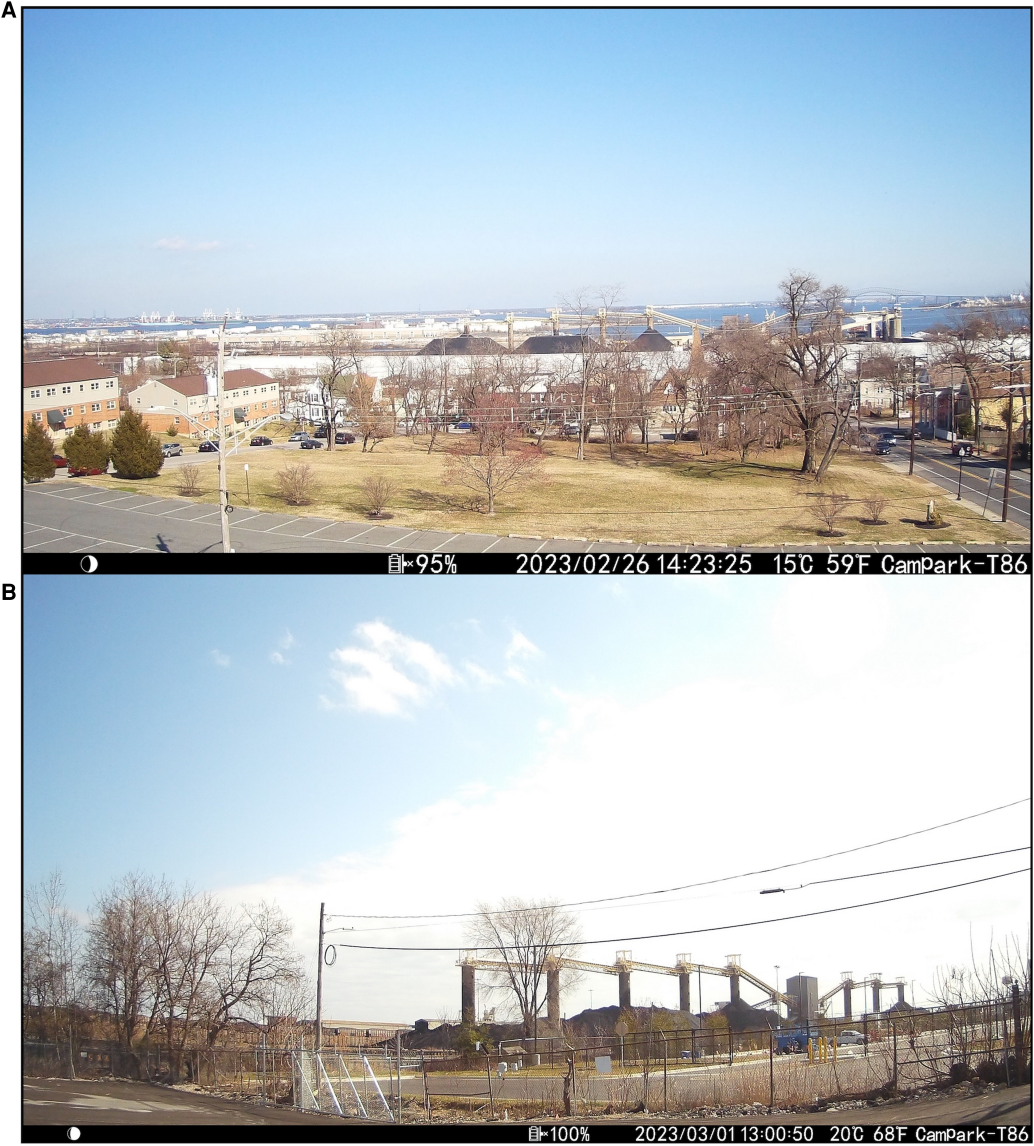
(**A**) Image of CSX Coal Terminal captured from the trail camera to the west of the terminal. (**B**) Image of CSX Coal Terminal captured from the trail camera to the north of the terminal.

Two CamPark T86 trail cameras (CamPark Electronics Co., Ltd, Hong Kong) are stationed at sites where the CSX Coal Terminal is clearly visible. The camera primarily used for data collection is located to the west of the coal terminal and has a full view of the facility from North to South (Figure 4A). The second camera is co-located with MODULAIR and ObservAir monitors near the northern edge of the facility (Figure 4B).

**Figure 5:**
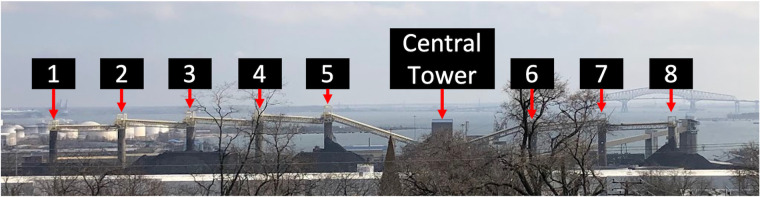
View of CSX Coal Terminal used for analysis of activity patterns (trains, ships, bulldozers, cranes, visible smoke or dust), presence of fog, and changes in coal pile dimensions (height and width). Tower numbers are used for orientation in analysis.

The day and night-vision trail cameras photograph the coal terminal every minute. Photos are stitched together into a time lapse video weekly. Academic research team members watch the time lapse videos and record terminal activity patterns (trains, ships, bulldozers, cranes, visible smoke or dust), coal pile height and width, and presence of fog (Tables 3, 4; Figure 5) as closely as possible to 1-minute resolution. The time lapse analysts conduct interrater agreement analysis by calculating percent agreement between five days of the same footage.

**Table 3:** Activities at the CSX Coal Terminal visually observed with trail cameras and transcribed using the described notation.

Activity/Variable	Notation
Trains present at the coal terminal	1 = trains present at coal terminal
	0 = no trains present at coal terminal
Trains entering the coal terminal premises	1 = trains traveling southbound into the coal terminal
	0 = no trains traveling southbound into the coal terminal
Trains leaving the coal terminal premises	1 = trains traveling northbound out of the coal terminal
	0 = no trains traveling northbound out of the coal terminal
Ships present at the coal terminal	1 = ships present at coal terminal
	0 = no ships present at coal terminal
Ships approaching or being loaded at the coal terminal	1 = ship approaching or being loaded at coal terminal
	0 = no ship approaching or being loaded at coal terminal
Ships leaving the coal terminal premises	1 = ship leaving the coal terminal
	0 = no ship leaving the coal terminal
Sprinkler system activated, spraying on the coal piles to minimize fugitive dust	1 = sprinkler system active
	0 = sprinkler system not active
Sprinkler system in operation north of the central tower	1 = sprinkler system in operation north of the central tower
	0 = sprinkler system not in operation north of the central tower
Sprinkler system in operation south of the central tower	1 = sprinkler system in operation south of the central tower
	0 = sprinkler system not in operation south of the central tower
Crane in operation at the coal terminal	1 = crane in operation
	0 = crane not in operation
Bulldozers in operation at the coal terminal	1 = bulldozers in operation
	0 = no bulldozers in operation
Bulldozers in operation north of the central tower	1 = bulldozers in operation north of the central tower
	0 = no bulldozers in operation north of the central tower
Bulldozers in operation south of the central tower	1 = bulldozers in operation south of the central tower
	0 = no bulldozers in operation south of the central tower
Visible smoke or dust originating from the coal terminal	1 = visible smoke or dust originating from the coal terminal
	0 = no visible smoke or dust originating from the coal terminal
Visible smoke or dust originating from north of the central tower	1 = visible smoke or dust originating from north of the central tower
	0 = no visible smoke or dust originating from north of the central tower
Visible smoke or dust originating from south of the central tower	1 = visible smoke or dust originating from south of the central tower
	0 = no visible smoke or dust originating from south of the central tower
Visible fog	1 = visible fog
	0 = no visible fog

**Table 4:** Description of notation used for height and width of every visible coal pile at the CSX Coal Terminal.

Variable	Notation
Height of coal pile at each visible stacking tower	1 = height of coal pile is at or above half the height of the central stacking tower
	0 = height of coal pile is below half the height of the central stacking tower
Width of the coal pile at each visible stacking tower	N/A = camera view of coal pile is obstructed
	“.” = no coal pile at stacking tower
	0 = coal pile width is <50% the distance to the adjacent tower
	1 = coal pile width is >50% to <100% of the distance to the adjacent tower
	2 = coal pile width is at or beyond adjacent tower
	3 = coal pile joins with an adjacent coal pile

Observations and testimonies from community team members and CB residents also provide context to air quality data. For example, community residents noted the presence of fog in the area when determination from trail camera footage was not possible. This information was incorporated into data quality control processes.

#### 2.5.3. Data analysis

We merge the air quality data and the transcribed CSX Coal Terminal activities/coal pile conditions by date and time to 1-minute resolution using R data analysis and statistical software (version 4.1.2) (59). We use the tidyverse (60) package for data cleaning and wrangling, while we use the ggplot2 (61) and openair (62) packages for data visualization of air quality and terminal activity trends. Using the combined dataset, the community-academic team evaluates overall air quality in CB, multi-air pollutant profiles of elevated events, spatiotemporal changes in air quality in the community, patterns of industrial activity, and potential correlations between air quality and observed activity. The team identifies probable sources of elevated air pollution events using the framework shown in Table 5 and via discussion with MDE and air monitor manufacturer representatives.

**Table 5:** Framework for identifying types of air pollution events.

Probable Type of Air Pollution Event	Pollutants Elevated in Signature
Fugitive coal dust emission	• PM_10_ and/or TSP • BC
Diesel combustion emissions	• PM_1_ and/or PM_2.5_ • BC • CO, CO_2_, and/or NO_x_
Non-combustion particulate matter	• PM_1_ and/or PM_2.5_
Road dust or other coarse-mode particulate matter	• PM_10_ and/or TSP

We maintain data quality using the following quality assurance and quality control approaches: co-locate MODULAIR and ObservAir monitors with regulatory (or regulatory-grade) monitors to develop correction factors, apply data cleaning criteria, discuss potential confounding factors (e.g., relative humidity and local fog events) with MDE technical team, and apply flags to adjust dataset for confounders.

QuantAQ has their own data cleaning and correction process for the MODULAIR which involves flagging data points where sensors have failed, correcting for aerosol density and environmental factors (e.g., hygroscopic growth) (for particles), and flagging the first hour of data after the monitor has re-started (for gases) (Dr. David H. Hagan, oral communication, June 23, 2023) (63, 64). In addition to these procedures, our data team has worked with MDE to establish a fog flag for PM_10_ and TSP data. This flag is “raised” when relative humidity is greater than or equal to 80%, PM_10_ concentrations are greater than or equal to 200 µg/m^3^, and when TSP concentrations are greater than or equal to 500 µg/m^3^. Flagged PM_10_ and TSP data are disregarded in the final dataset.

DSTech also accounts for changes in relative humidity and temperature in their data processing (65). Further, there are also corrections applied as the sampling filter becomes saturated and light attenuation decreases (66–69). Finally, DSTech employs a digital low pass filter with the goal of “decreas[ing] measurement noise” (70). We cleaned ObservAir BC data by disregarding measurements when they are equal to −5 µg/m^3^, when there are low battery voltage readings (battery voltage less than or equal to 7.5 volts), during the first hour after the monitor has been off for more than 15 min, and when there are rapid changes in relative humidity with respect to time (when there is a 5-minute absolute instantaneous change in relative humidity greater than 0.03% while BC concentrations are <–0.4 µg/m^3^ or BC concentrations are −5 µg/m^3^).

### 2.6. Engaging youth in local environmental justice efforts: capacity building and community organizing

A core component of our approach is to establish this hyperlocal network with project sustainability and community capacity building as a priority. Team members from CSI EJ and Towson University (TU) co-lead an EJ course at BFHS which is a continuation of FYV and a partnership that TU has cultivated for five years. Interested students in the 11th and 12th grades submitted applications and were selected by BFHS teachers/staff to participate in a semester-long course where they earn community service hours and three college credits. We utilize an iterative process for the course curriculum and activities, responsive to students' interests and research questions.

Students are introduced to the long history of environmental injustices and community-led victories in CB. They are taught principles and practices of community science, applying methods of five-sense ethnography, and recording scientific observations. The team also instructs about how to utilize both qualitative and quantitative data collection activities to contribute to the local EJ movement. For the first component of their final project, the students developed and administered a school-wide survey about youth perceptions of environmental injustice in their communities. This survey is a precursor to a larger health panel study planned by the community-academic team. In the second component of the final project, the students analyzed air quality data from a MODULAIR air monitor deployed at their school (Location 9 in Figure 3). They have developed dissemination products conveying their new understanding of criteria air pollutants, EJ concerns in CB, and their own data visualizations of air quality at their school. Students are invited to use their new skills to join ongoing EJ work in the community and continue their training.

## 3. Results and discussion

### 3.1. Agreement between co-located air monitors

We conducted two types of co-locations: regulatory and intercomparison. This was done for four pollutants: BC, PM_2.5_, PM_10_, and CO. Regulatory co-locations took place at sites with regulatory (Howard County Near Road, MD) or regulatory-grade instruments (Pocomoke City, MD), while the intercomparisons took place at Location 8 in the CB hyperlocal air monitoring network (Supplementary Table S1). When comparing low-cost sensors to regulatory monitors, we observed good agreement for all pollutants except PM_10_. Upon further data exploration, we plan to use these results to calculate correction factors for our data. For the low-cost sensor intercomparisons, we observed excellent agreement.

Please see Supplementary Material and Supplementary Figures S1–5 for information about agreement between air pollutants (BC, PM_2.5_, PM_10_, and CO) using regulatory or regulatory-grade monitors at MDE Ambient Air Monitoring Network sites and from co-located deployments of our QuantAQ MODULAIR and DSTech ObservAir LCSs. For intercomparison between LCSs, please see Supplementary Figures S6–9.

### 3.2. Ground-truthing example: industrial fire event in Curtis Bay

The hyperlocal network and ground-truthing methods have demonstrated data-driven responsiveness to emergent community concerns in addition to informing about overall air quality.

**Figure 6:**
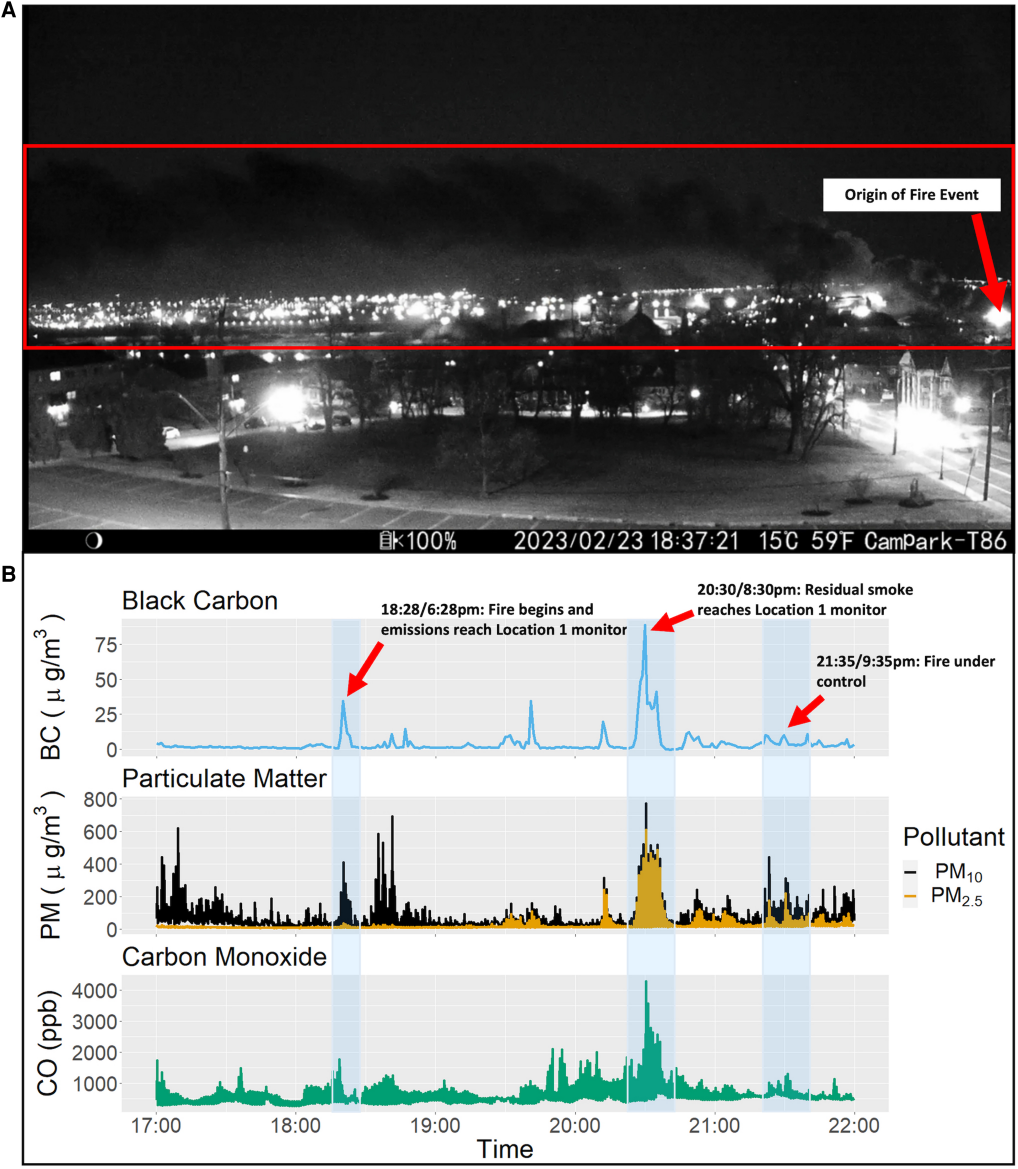
(**A**) Still image from trail camera time lapse video of visible smoke from the industrial fire. Plume of smoke blowing north (right to left) shown in red box. (**B**) Time series of black carbon (BC), particulate matter (PM_2.5_ and PM_10_), and carbon monoxide (CO) during the fire event.

An industrial fire occurred on February 23, 2023, at approximately 6:30 PM EST near Location 1 of our network (Figure 3, Supplementary Video S1). The industrial area where the fire occurred is a few hundred meters away from CB residences. Community residents asked SBCLT and CCBA for information about the fire as they took their children home from the nearby community recreation center. The community-academic team then utilized the ground-truthing approach to pinpoint visual observation of the fire using trail camera footage (Figure 6A, Supplementary Video S1) and a parallel multi-pollutant signature of the event (Figure 6B). The MODULAIR and ObservAir at Location 1 detected simultaneous elevated levels of BC (µg/m^3^), PM_2.5_ and PM_10_ (µg/m^3^), and CO (ppb). Community resident observations and information about the event from the MDE team assisted in identifying the causes of each significant pollutant spike during the fire event (Figure 6B).

The visuals in Figure 6 were included in a presentation to community residents about the fire event and the community-driven hyperlocal air monitoring network. This is an example of how our network can provide real-time data about elevated air pollution events which can be used for raising community awareness and regulatory or enforcement action.

### 3.3. Best practices for community-academic partnerships in communities facing similar environmental justice concerns

Equitable and committed community-academic partnerships can be a powerful driver for empowerment and achieving improved health outcomes. Our team has developed a list of best practices and recommendations for other partnerships seeking to address local EJ concerns.

#### 3.3.1. From our community partners to other community-academic partnerships

• Commit time, energy, and resources to listening to residents and workers at every stage of the EJ research and action process.
• Evaluate governance structures of local community-based organizations with a focus on resident/worker participation.
• Honor what residents share and describe and resist the tendency to translate concerns or redirect them out of convenience or a sense of pragmatism.
• Make connections and look for patterns in what you are hearing. Actively work against the divisions and isolating effects that conditions in EJ communities create by facilitating dialogue and connections between residents and groups.
• Trust the direct experience of residents as well as the capacity of residents to take on significant responsibilities and develop new capacities. Residents are the experts! Center a belief in community-led research.
• Seek connections with communities facing similar challenges…realize you are not alone in your struggle.
• Anticipate a seemingly infinite number of challenges and roadblocks by building in staying power for your effort. Recognize and celebrate any breakthroughs at any level.
• Emphasize the multigenerational nature of the challenges being taken on. Affirm the possibility of success but do not pin the wellbeing of the effort on realizing it in a short time frame.

#### 3.3.2. From our academic partners to other academic partners

• Be intentional about prioritizing community needs and input.
• Spend time cultivating relationships; trust is key to doing this work successfully.
• Openly commit to long-term engagement from the start of the partnership. The community partners and residents experience environmental injustice daily and, likely, have for multiple generations. Committing and adhering to long-term collaboration from the outset builds trust, accountability, and informed context to scientific findings.
• Understand and acknowledge your institution's historical relationship with the community and the ways in which it has harmed the community.
• Be transparent about what you can bring to the relationship and your limitations.
• Come to the community with humility and understand that building trust takes time and effort. Academic or scientific and technical knowledge is not the end-all-be-all; the lived experience of the community you are working with is just as, if not more, important in advancing solutions to community-identified needs.
• Reject expectations of a transactional relationship with your community partners. In a transactional relationship, each partner prioritizes individual objectives which upholds an “us” vs. “them” power dynamic (71, 72). Transactional relationships cannot coexist with long-term, equitable partnerships and have no place in meaningful community-academic collaboration. Emphasis should be placed upon the co-creation of knowledge, co-learning, and shared objectives of the partnership.
• Intentionally center anti-racist, anti-classist, and inclusive ideologies and practices in your research (73) by:
  ⚬ Explicitly acknowledging historical, systematic, and individual racism, classism, and discrimination as drivers of the social, health, and environmental inequities affecting EJ communities;
  ⚬ Honoring the perspectives and lived experiences of residents in EJ communities by having marginalized groups empowered in knowledge creation, leadership, and decision-making;
  ⚬ Assisting community partners in obtaining funding to support their work outside the scope of your research;
  ⚬ Developing research objectives toward meaningful and equitable action while being flexible to amend your aims and perspectives in line with community priotities.

The air monitoring network described here has brought greater attention to the CB neighborhood and the living EJ movement in the community. Amidst increased interest and scrutiny, adhering to the above tenets has helped our team to remain united in our commitment to improving health and quality of life in CB while empowering community members' lived experience. Although the EJ issues and the community vision may differ in other contexts, adhering to these best practices and developing unique and explicitly shared values keeps both groups accountable to one another and to the broader community.

### 3.4. Challenges, successes, and next steps

Through this ongoing community-driven air monitoring and capacity building effort, our team has run into several challenges and celebrated our successes, while planning for future steps to expand and improve our work. Our reflection upon these experiences will hopefully aid other communities facing similar EJ concerns and considering similar approaches.

#### 3.4.1. Challenges faced

From experiences with academic groups in the past, our community partners identified researchers' tendencies to view issues in siloes, rather than recognizing the interconnectedness of communities and environmental injustices. For example, the uncovered train cars carrying coal to the CSX Coal Terminal in CB cut through other South Baltimore communities. Residents in these low-income and historically disinvested neighborhoods also have reported dark—presumably coal—dust on exterior and interior surfaces of their homes. In recognizing the aforementioned academic tendency, one of our core objectives was to be posed to expand into interested neighboring communities sharing EJ concerns.

Data quality is a common concern when using low-cost air monitors. Therefore, data quality assurance and control are critical in our community-engaged air monitoring network, as evidenced by our extensive data cleaning and co-location procedures. This presents an important tradeoff, also evident in other forms of CEnR: the time required to employ traditional protocols and improve data quality can be incongruent with and unresponsive to community needs for dissemination and external timelines (e.g., industrial operation permit renewal deadlines). However, having community members as participants in these processes fosters mutual understanding and co-learning about how to balance this tradeoff. Our community-academic team simultaneously worked through data quality control and assurance with MDE while outreaching with other community members and organizations.

Simultaneous activities, on the other hand, also create a lack of attention to some items over time. For an example from our work, MDE required the CSX Coal Terminal to improve their fugitive dust control plan and develop a new fenceline monitoring plan in response to the 2021 explosion and decades-long complaints from South Baltimore residents. Our community-academic team developed public commentary for both plans, merging community experiences with scientific support. With focus and effort turning towards the community-driven hyperlocal air monitoring network over time, follow-up regarding these plans became less frequent and we imposed less accountability upon MDE and CSX to follow through.

#### 3.4.2. Successes celebrated

Nonetheless, the improvement and development of the CSX Coal Terminal fugitive dust control and fenceline monitoring plans stem from the action of our community partners and the support of our community-academic partnership—a major success. This demonstrates our ability to hold polluters and regulators accountable and the power of having community at the table in all aspects of our work. The direct line of communication between CB community representatives and the MDE Air and Radiation Administration has allowed for improved public communication about industrial emergencies. For example, CB residents learned of a chemical spill at a chemical manufacturing and processing plant in May 2023, raising justified alarm in community chats and social media. Through urgent meetings and correspondence between our community-academic team and MDE, CB community representatives received detailed information directly from local regulators about possible environmental hazards and the gravity of the concern. Our partners then updated other CB residents with developing information.

Our capacity building and youth education efforts are expanding into a summer fellowship for high school students and recent graduates, with the first cohort in the summer of 2023. Summer fellows will become more deeply engaged in the local EJ movement through ongoing activism and supporting community science initiatives, such as our hyperlocal air monitoring network and health communication. Fellows will be compensated for their participation and receive valuable skills to become future environmental justice and health leaders. The inaugural cohort will be invited the following summer to support the fellowship as mentors and trainers, ushering in the next generation of EJ trailblazers.

Acknowledging and embracing interconnectedness in our community-driven work is a major success and continuous, intentional endeavor. Embracing interconnectedness began in our objectives with building applicability to other South Baltimore communities. We have begun to partner with other community associations to deploy LCSs and join the community-level air monitoring network. In time, we hope our network can provide powerful, actionable data about air quality and sources of pollution throughout South Baltimore. Community members will continue to guide our objectives and placement of monitoring sites as this network expands.

Interconnectedness has also developed within the CB context, particularly with activism. Action coalitions have mobilized around similar efforts addressing the CSX Coal Terminal and broader community concerns with health and safety due to industrial activity. Members of our research team have participated in community demonstrations and rallies in defiance of industrial encroachment in the community. Preliminary data from our community-academic partnership and hyperlocal air monitoring network have helped to support community testimonies and empower activism.

#### 3.4.3. Future steps

A significant next step for our community-driven hyperlocal air monitoring network will be to add new measured pollutant parameters. Air pollutants of concern in CB include volatile organic compounds and air toxics which are not currently addressed in the low-cost sensors in use in our network. In response to community requests and acknowledging uncharacterized air pollution in CB, we will be exploring monitoring and sampling methods for volatile organic compounds in the near future.

As the breadth of our air monitoring network expands, so will our need for personnel to maintain and operate the network long-term. Our community-academic team will be ramping up shadowing and training activities for local youth and other community members. In time, our work will evolve from community-driven to community-owned and -operated, encompassing data collection, data analysis, and data dissemination. Near-term actions include creating standard operating procedures for air monitors and trail cameras with video instruction and available R code for data cleaning and analysis.

Future steps also include improving our dissemination of findings from our air monitoring network. We have begun to develop weekly air quality summaries for our monitor site hosts and community organizations using the openair package in R (62). Weekly summaries allow our monitor hosts to discuss any observations of activities that may have contributed to elevated air pollution. Improvements to our data quality assurance/control and analysis processes will augment the strength and validity of our data. For example, we can use wind speed and wind direction to estimate possible lag time between camera-observed activities and air quality measurements. We will also apply *a posteriori* correction factors to collected LCS data, accounting for bias and unknown factors.

Our team will also conduct a community health panel study around the potential health effects of community exposure to ambient and settled coal dust. Participants will conduct daily symptom and quality of life diaries with concurrent air monitoring and surface dust sampling at their home. This participatory and community-engaged health panel will also be a training opportunity for summer fellows and an element of cross-community engagement. Participant geographic eligibility will be dictated by the shared concern of coal dust and coal-related infrastructure (e.g., coal transport trains and railways) throughout South Baltimore communities.

Our relationship with the MDE Air and Radiation Administration has been critical to the success and evolution of this community-driven hyperlocal air monitoring network. In the short-term, we hope data collected will inform and strengthen industrial operation permits which require community and public input. As decision-makers at MDE become increasingly familiar with residents in the CB community and the EJ issues they experience—alongside robust quantitative air pollution data—regulation and enforcement can better protect EJ communities like CB. In the long-term, these improved environmental regulatory and enforcement decisions can drive structural and systematic change with ramifications in communities across Maryland and the US.

## 4. Conclusion

In this paper, we outlined our approach to addressing longstanding community-identified EJ issues using scientific/technical strategies and capacity building. Our hyperlocal air monitoring network continues to collect real-time air pollution data, observe industrial activity, and identify possible correlations to characterize the effect of industrial activity upon air quality in CB. The capacity building component of our approach acknowledges and enacts the need for long-term sustainability, co-learning, and community empowerment in EJ efforts. High school students are provided with college credits and community service hours as they learn about quantitative and qualitative data collection strategies. Next steps include growing and fine-tuning our network and data analysis procedures, a community health panel study to characterize community health outcomes and air pollution exposure, and continuing to engage youth leaders in the local EJ movement.

Our community-academic partnership continues to learn from one another and evolve our community-driven approach as we work together towards just community development in CB and South Baltimore.

## Data Availability

The raw data supporting the conclusions of this article will be made available by the authors, without undue reservation.
