# Supplementary material for: Community-driven research and capacity building to address environmental justice concerns with industrial air pollution in Curtis Bay, South Baltimore
Source: Front Epidemiol. 2023 Sep 12;3:1198321. doi: 10.3389/fepid.2023.1198321 (PMC10720608; doi:10.3389/fepid.2023.1198321)
Supplement: Supplementary file 2 [file Datasheet1.docx]

Supplementary Material

Community-driven research and capacity building to address environmental justice concerns with industrial air pollution in Curtis Bay, South Baltimore

Matthew A. Aubourg, Greg Sawtell, Lauren Deanes, Nicole Fabricant, Meleny Thomas, Kristoffer Spicer, Caila Wagar, Shashawnda Campbell, Abigail Ulman, Christopher D. Heaney*

*** Correspondence:** Christopher D. Heaney: cheaney1@jhu.edu

# Supplementary Video of Ground-Truthing Example Fire Event from February 23, 2023

#
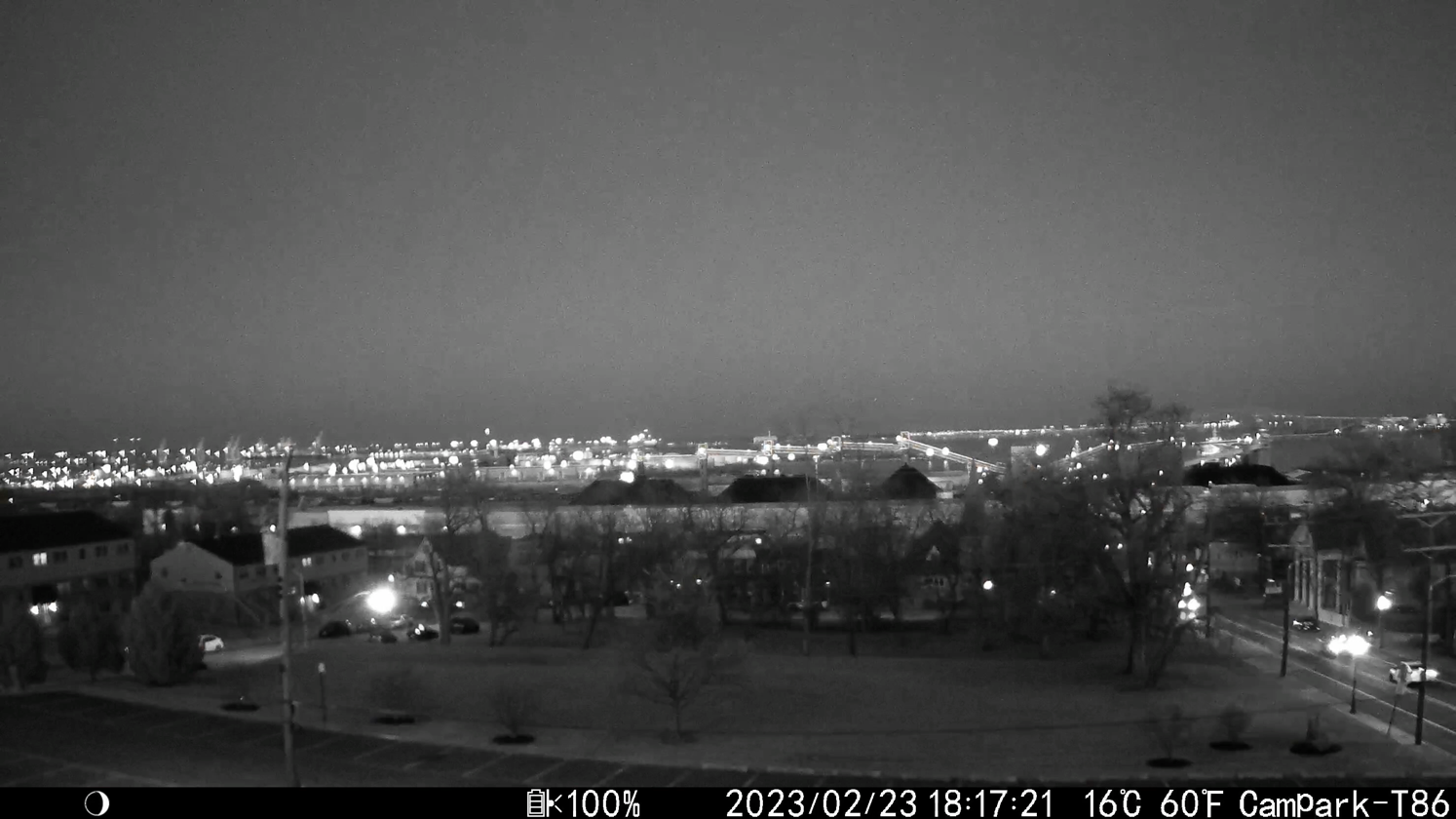


**Supplementary Video 1.** Time-lapse video created from trail camera images during a fire at an industrial facility in Curtis Bay, MD. Smoke from the fire is visible from the right side of the frame beginning from time stamp 00:02.

# Air Monitor Co-location Methods

We conducted co-locations of two types, regulatory and intercomparison. During regulatory co-locations, we deployed low-cost sensors (Distributed Sensing Technologies [DSTech] ObservAir and QuantAQ MODULAIR) at the Howard County Near Road (HCNR) and Pocomoke City sites in Maryland Department of the Environment (MDE) Ambient Air Monitoring Network (1). During intercomparison co-locations, we assessed agreement between two low-cost sensors of the same type. The duration of each co-location spanned from 14 to 29 days, and we explored agreement for the following pollutants: black carbon, PM_2.5_, PM_10_, and carbon monoxide. Further details about the duration of co-location, monitors used, and pollutants measured can be found in Supplementary Table 1.

**Supplementary Table 1.** Description of Co-Location Deployments.

| Co-location Type | Site | Duration | Pollutant | Low-Cost Sensor | Regulatory / Regulatory-Grade Monitor |
| --- | --- | --- | --- | --- | --- |
| Regulatory / Regulatory-Grade | Howard County Near Road (HCNR), MD^a^ | 14 days | Black Carbon^c^ | Distributed Sensing Technologies ObservAir | Magee Scientific Aethalometer AE33 |
|  |  |  | PM_2.5_ | QuantAQ MODULAIR | Met One Continuous Particulates Monitor BAM 1020 |
|  |  |  | Carbon Monoxide |  | Teledyne API T300U |
|  | Pocomoke City, MD^b^ | 14 days | PM_2.5_ | QuantAQ MODULAIR | Teledyne API 640X |
|  |  |  | PM_10_ |  |  |
| Intercomparison | Curtis Bay Network Location 8 | 29 days | Black Carbon | Distributed Sensing Technologies ObservAir | N/A |
|  |  | 22 days | PM_2.5_ | QuantAQ MODULAIR |  |
|  |  |  | PM_10_ |  |  |
|  |  |  | Carbon Monoxide |  |  |

^a^Regulatory site part of the MDE Ambient Air Monitoring Network (1); ^b^Site with regulatory-grade instrument part of the Lower Eastern Shore Ambient Air Quality Monitoring Project (2); ^c^Black carbon is not a “criteria air pollutant,” (3) but MDE collects black carbon measurements using “special purpose monitors,” such as the Magee AE33 (1).

To assess agreement for each co-location, we performed ordinary least squares simple linear regression. As suggested by the 2022 EPA Enhanced Air Sensor Guidebook, for regulatory comparisons, the low-cost sensor was the dependent variable and the regulatory monitor was the independent variable for each of those regressions (4). Further, we calculated Pearson r correlations (and their associated p-values), the adjusted coefficient of determination (R^2^), and the root-mean-square error (RMSE) for each regression model.

# Air Monitor Co-location Results

## Comparison of Low-cost Sensors and Regulatory Instruments

First, for black carbon, we observed a very strong and positive correlation between the Magee AE33 monitor and the ObservAir at HCNR (Pearson r = 0.95, p-value < 0.005) (Supplementary Figure 1). These findings are consistent with field validation conducted by DSTech, where Pearson r correlations (when comparing six ObservAirs to a regulatory instrument) ranged from 0.89 to 0.95 (5).

Secondly, the PM_2.5_ co-location yielded strong correlations for both sites. At HCNR, there was a positive and strong correlation between the MODULAIR and Met One BAM 1020 (Pearson r = 0.78, p-value < 0.005) (Supplementary Figure 2). Like the findings for black carbon, the correlation was very strong and positive for PM_2.5_ at Pocomoke City (Pearson r = 0.90, p-value < 0.005). Supplementary Figure 3 illustrates that PM_2.5_ agreement at Pocomoke City is decent until higher concentrations, when the Teledyne 640X registered higher concentrations than the MODULAIR. This is consistent with EPA findings that the Teledyne 640X may be more likely to overestimate PM_2.5_ at higher concentrations, and this may explain the lower, albeit good, model fit (R^2^ = 0.812) (6).

Thirdly, we had access to PM_10_ data at only one regulatory-grade site (Pocomoke City, MD). We observed a moderate positive correlation between the regulatory-grade monitor (Teledyne API 640X) and the MODULAIR for PM_10_ (Pearson r = 0.56, p-value < 0.005), poor model fit (R^2^ = 0.31), and high RMSE (31.5 µg/m^3^) at that location (Supplementary Figure 4). The R^2^ value is lower than what QuantAQ has found when comparing hourly PM_10_ measurements from a MODULAIR to hourly measurements from a regulatory-grade monitor (R^2^ = 0.899) (7). Further, we also note the clustering in Supplementary Figure 4. Most concentrations from the Teledyne are below 50 µg/m^3^ while the same data points are bounded by 250 µg/m^3^ for the MODULAIR. This comparison warrants further study.

Finally, for carbon monoxide, we observed a very strong and positive correlation between the regulatory monitor at HCNR and the MODULAIR (Pearson r = 0.91, p-value < 0.005) (Supplementary Figure 5). Further, the model fit was good (R^2^ = 0.828); while RMSE was a bit high (42 ppb), it appears typical for these comparisons (Dr. David H. Hagan, oral communication, June 23, 2023). According to the 2014 EPA Air Sensor guidebook, our monitoring network fits into Tier II: “Hotspot Identification and Characterization” (8). The EPA suggested that for Tier II low-cost sensors, “a bias and precision of ± 30% might be reasonable.” The linear regression model suggests that carbon monoxide concentrations from the MODULAIR fit this criterion.

## Intercomparison between Low-cost Sensors

Pearson r correlations for all intercomparisons at Location 8 were excellent (ObservAir black carbon: Pearson r = 0.946, p-value < 0.005; MODULAIR PM_2.5_: Pearson r = 0.978, p-value < 0.005; MODULAIR PM_10_: Pearson r = 0.96, p-value < 0.005; MODULAIR carbon monoxide: Pearson r = 0.99, p-value < 0.005). Further, the model fits were very good, with R^2^ values of at least 0.89 for the four intercomparisons. For PM_2.5_, measurements are very close to 1:1 agreement (Supplementary Figure 7). See Supplementary Figures 6-9 for scatterplots of data comparing low-cost sensors.

# Air Monitor Co-location Figures


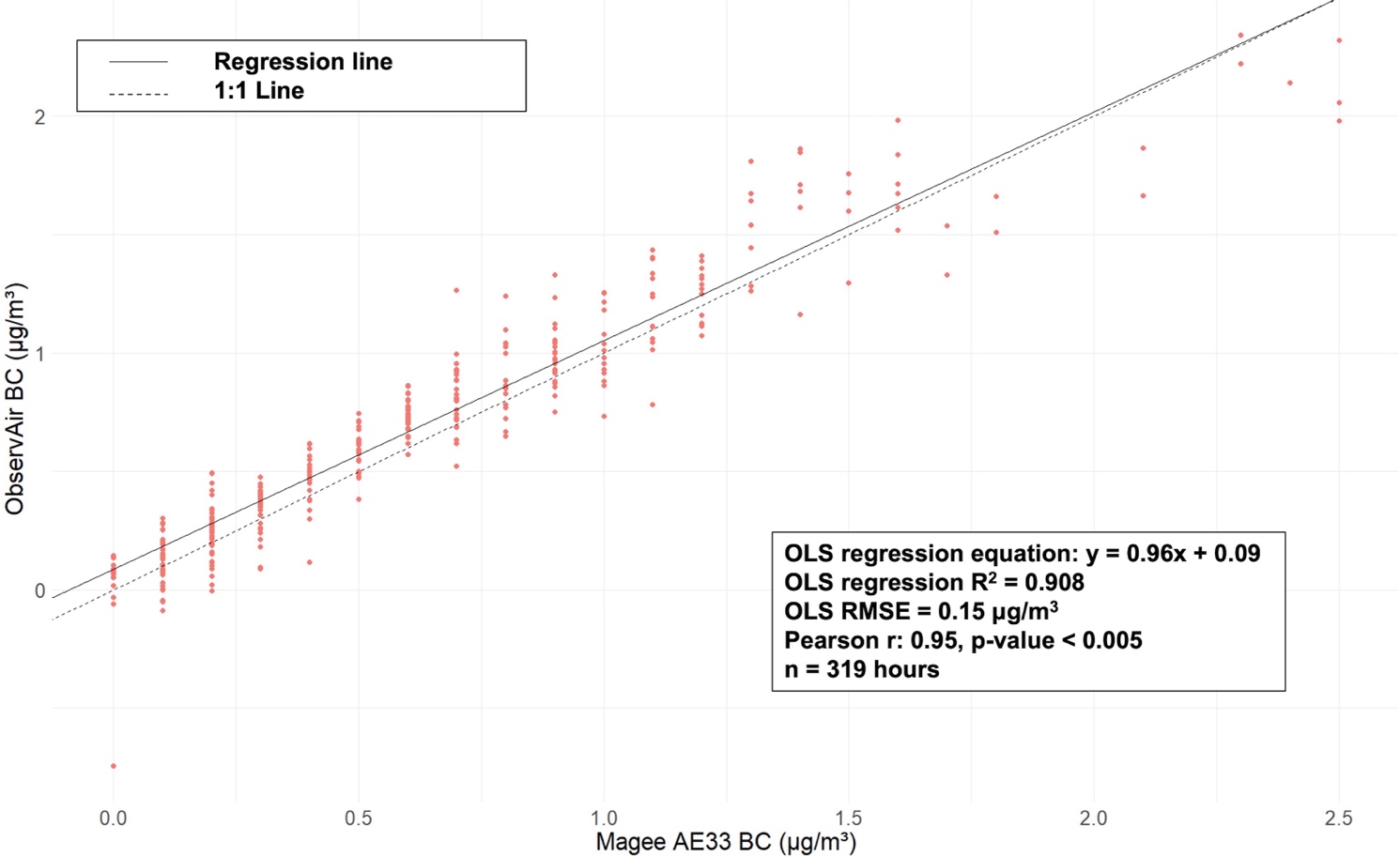


**Supplementary Figure 1.** Comparison between co-located, hourly-averaged Distributed Sensing Technologies ObservAir (low-cost sensor) and Magee Scientific Aethalometer AE33 (regulatory agency monitor) black carbon (BC) measurements (µg/m^3^) at the Howard County Near Road site in the Maryland Department of the Environment Ambient Air Monitoring Network (14-day co-location). *Note*: OLS = Ordinary least squares; in the OLS regression model, ObservAir BC is the dependent variable, *y*, and Magee AE33 BC is the independent variable, *x*. RMSE = root-mean-square error. n = sample size.


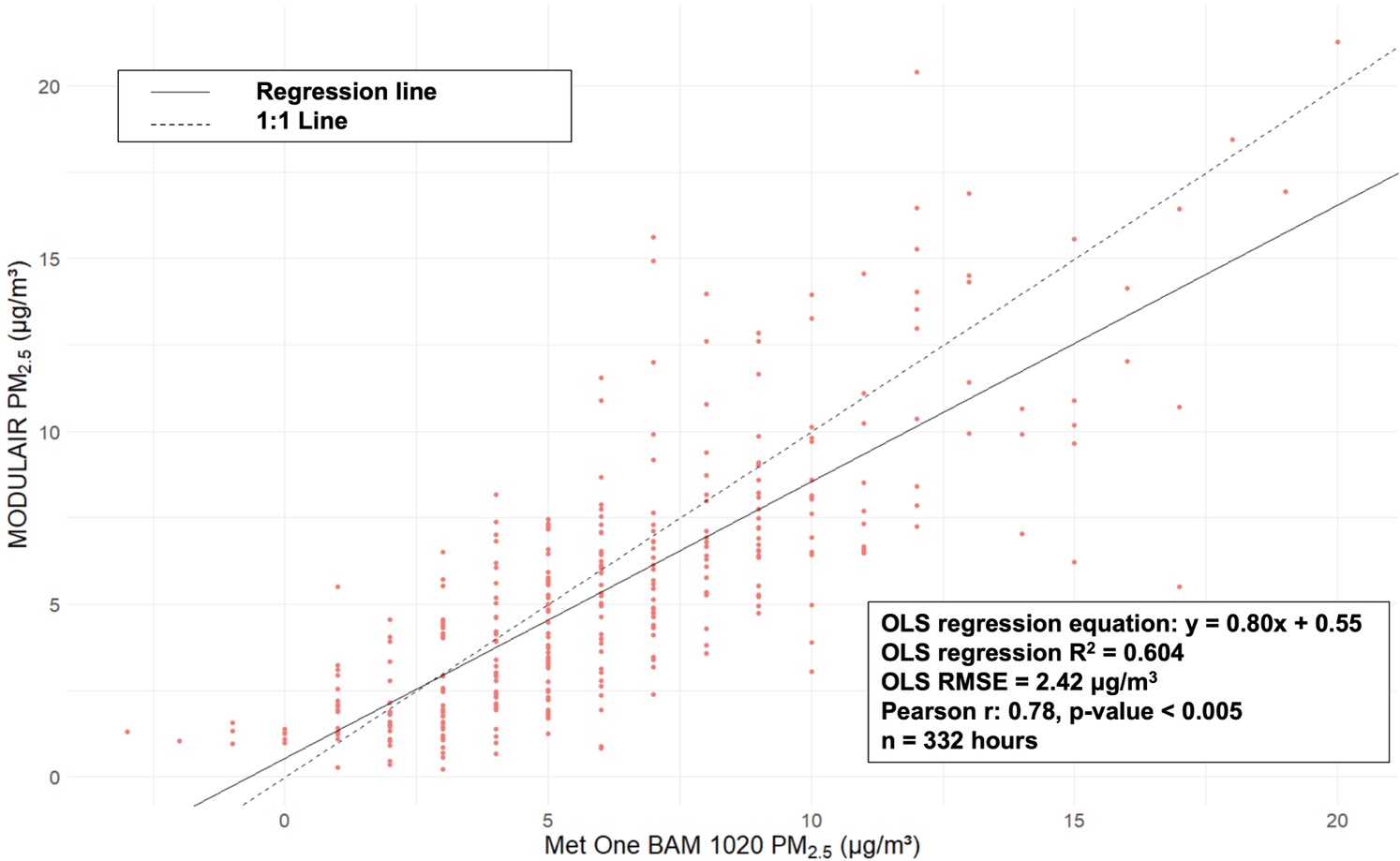


**Supplementary Figure 2.** Comparison between co-located, hourly-averaged QuantAQ MODULAIR (low-cost sensor) and Met One Instruments Continuous Particulate Monitor BAM 1020 (regulatory monitor) PM_2.5_ measurements (µg/m^3^) at the Howard County Near Road site in the Maryland Department of the Environment Ambient Air Monitoring Network (14-day co-location). *Note*: OLS = Ordinary least squares; in the OLS regression model, MODULAIR PM_2.5_ is the dependent variable, *y*, and Met One BAM 1020 PM_2.5_ is the independent variable, *x*. RMSE = root-mean-square error. n = sample size.


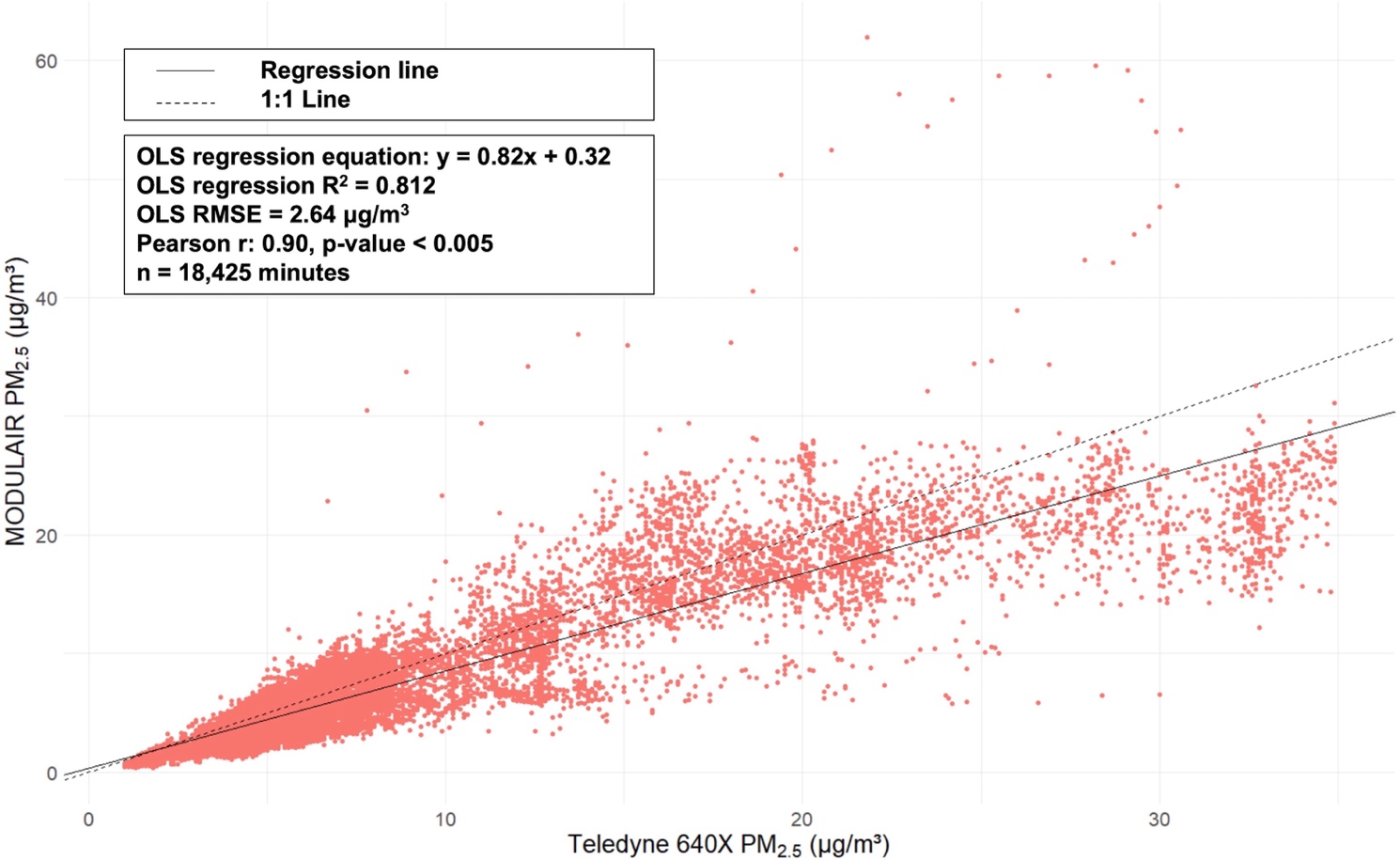


**Supplementary Figure 3.** Comparison between co-located, minute-averaged QuantAQ MODULAIR (low-cost sensor) and Teledyne API 640X (regulatory-grade monitor) PM_2.5_ measurements (µg/m^3^) at the Pocomoke City site in the Lower Eastern Shore Ambient Air Quality Monitoring Project (14-day co-location). *Note*: OLS = Ordinary least squares; in the OLS regression model, MODULAIR PM_2.5_ is the dependent variable, *y*, and Teledyne API 640X PM_2.5_ is the independent variable, *x.* RMSE = root-mean-square error. n = sample size.


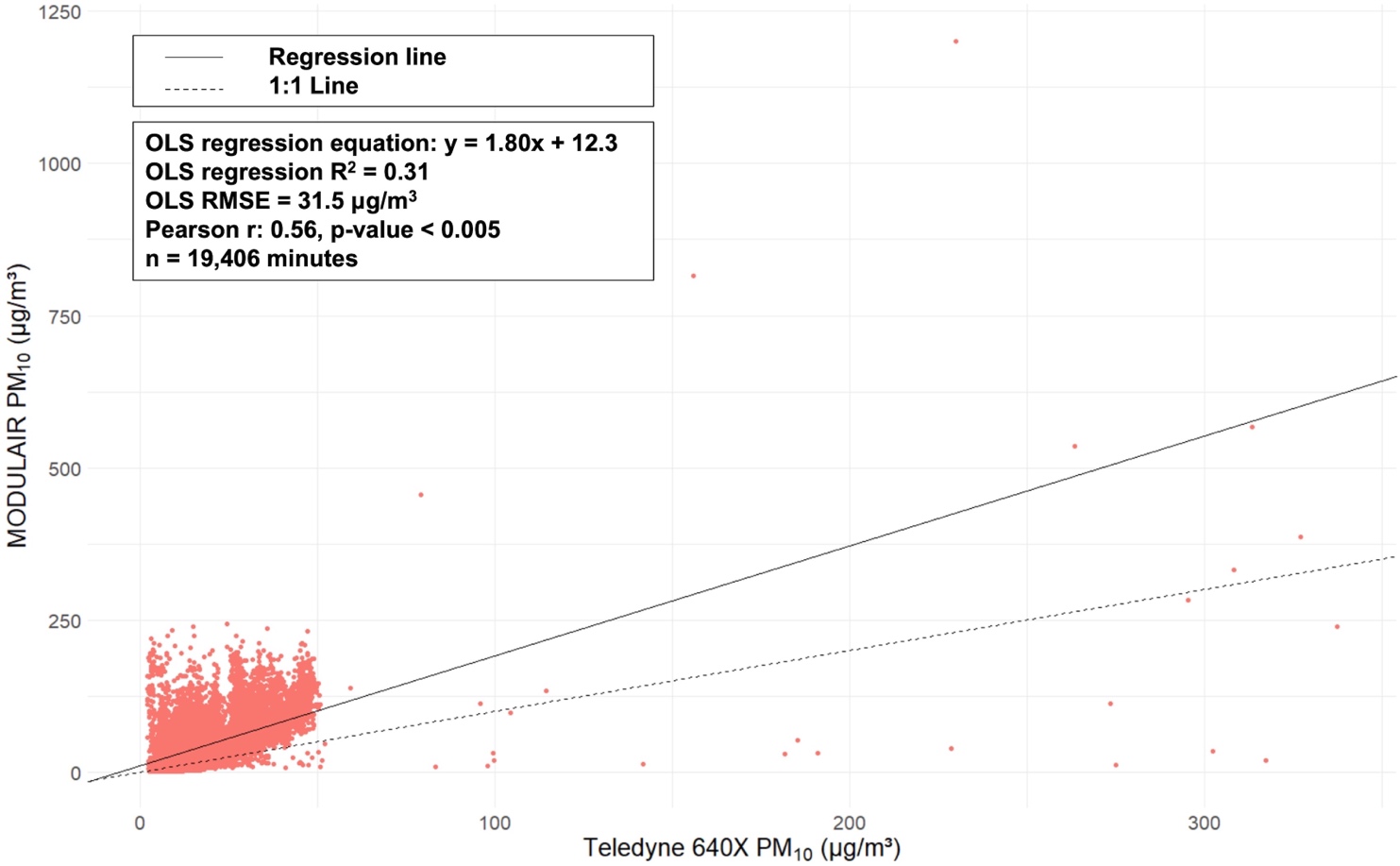


**Supplementary Figure 4.** Comparison between minute-averaged QuantAQ MODULAIR (low-cost sensor) and Teledyne 640X (regulatory-grade monitor) PM_10_ measurements (µg/m^3^) at the Pocomoke City site in the Lower Eastern Shore Ambient Air Quality Monitoring Project (14-day co-location). *Note*: OLS = Ordinary least squares; in the OLS regression model, MODULAIR PM_10_ is the dependent variable, *y*, and Teledyne API 640X PM_10_ is the independent variable, *x*. RMSE = root-mean-square error. n = sample size.


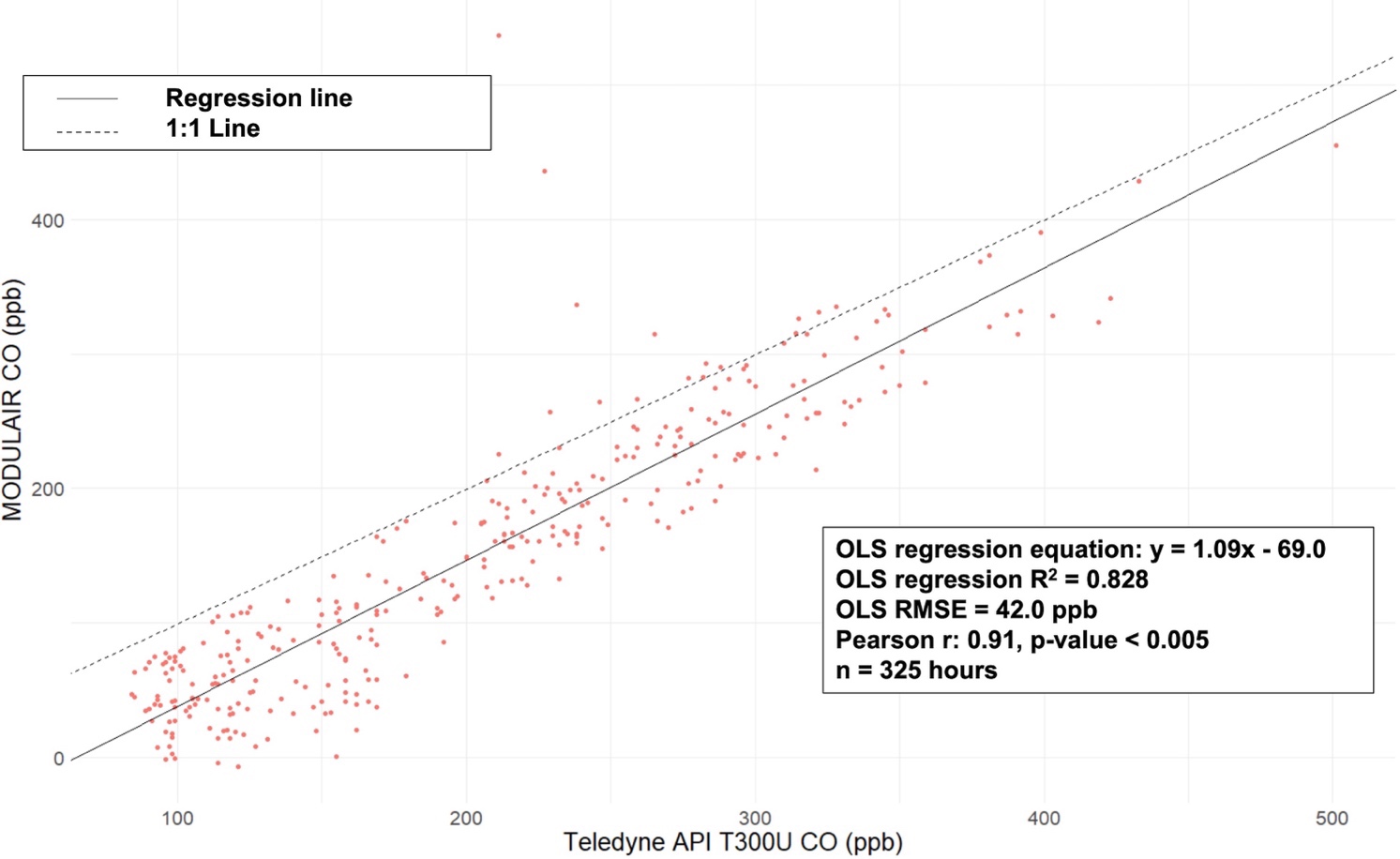


**Supplementary Figure 5.** Comparison between hourly-averaged QuantAQ MODULAIR (low-cost sensor) and Teledyne API T300U (regulatory monitor) carbon monoxide (CO) measurements (ppb) at the Howard County Near Road site in the Maryland Department of the Environment Ambient Air Monitoring Network (14-day co-location). *Note*: OLS = Ordinary least squares; in the OLS regression model, MODULAIR CO is the dependent variable, *y*, and Teledyne API T300U CO is the independent variable, *x.* RMSE = root-mean-square error. n = sample size.


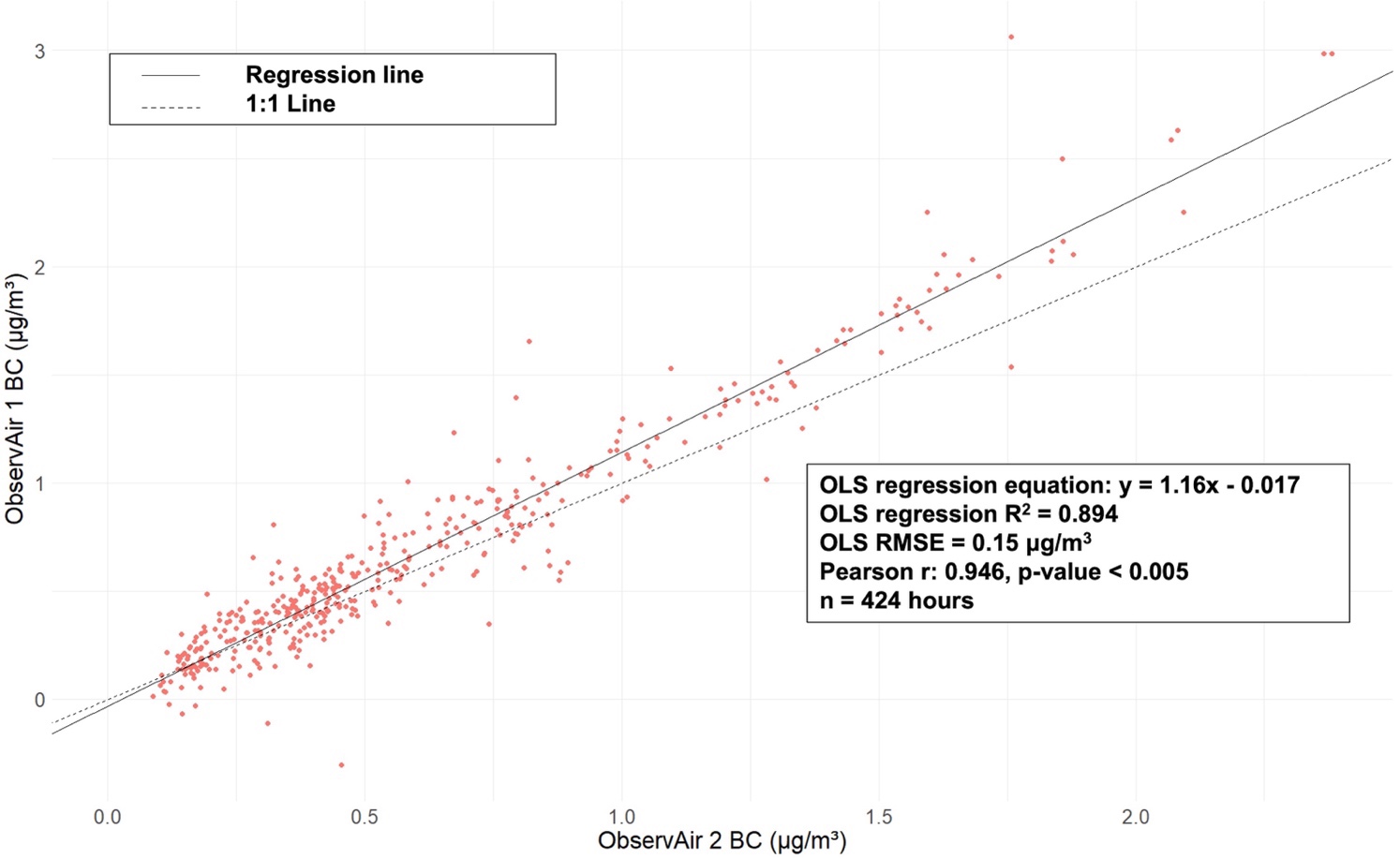


**Supplementary Figure 6.** Comparison between hourly-averaged Distributed Sensing Technologies ObservAir (low-cost sensor) black carbon (BC) measurements (µg/m^3^) at Location 8 in the Curtis Bay Hyperlocal Air Monitoring Network (29-day co-location). *Note*: OLS = Ordinary least squares; in the OLS regression model, ObservAir 1 BC is the dependent variable, *y*, and ObservAir 2 BC is the independent variable, *x*. RMSE = root-mean-square error. n = sample size.


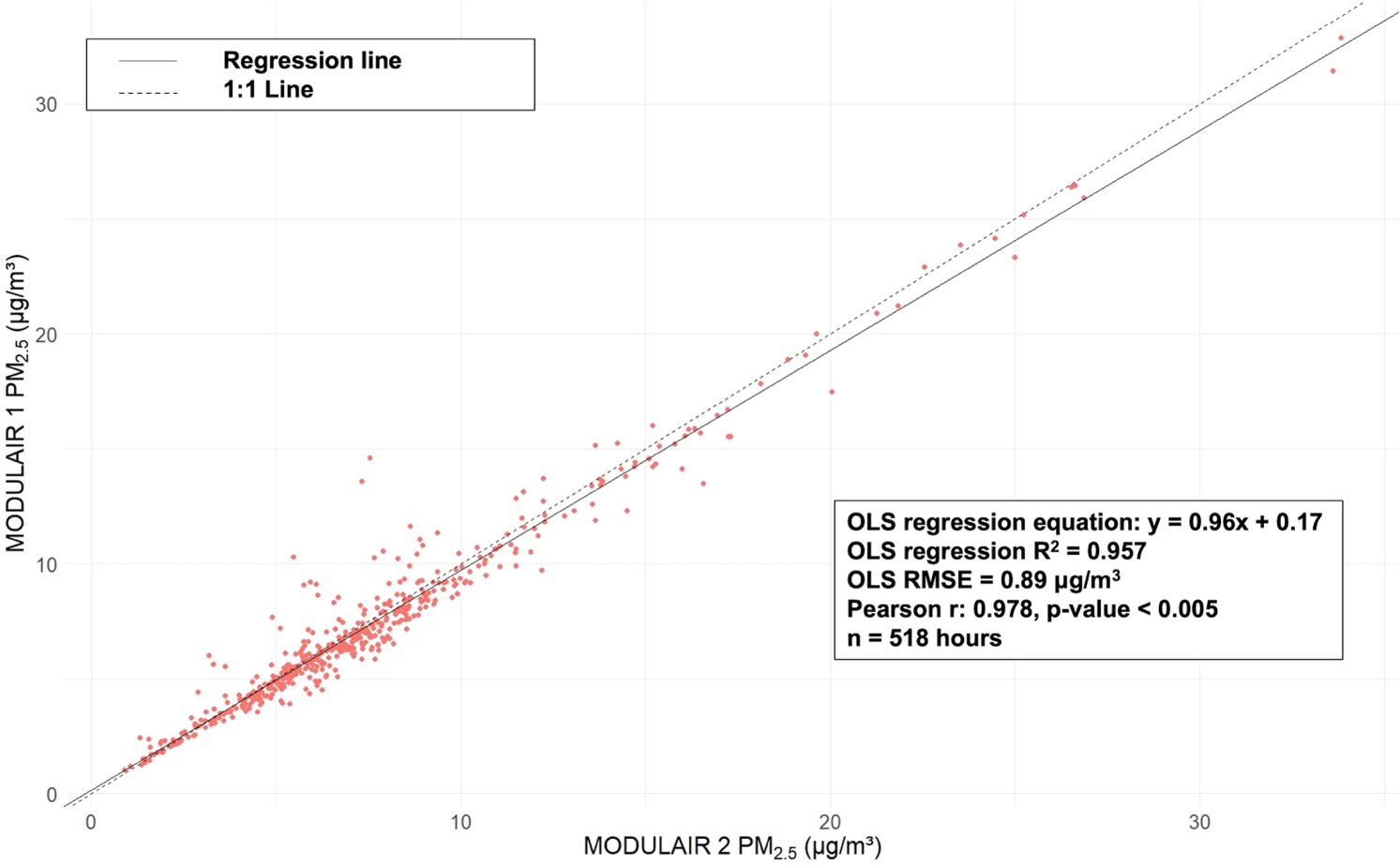


**Supplementary Figure 7.** Comparison between hourly-averaged QuantAQ MODULAIR (low-cost sensor) PM_2.5_ measurements (µg/m^3^) at Location 8 in the Curtis Bay Hyperlocal Air Monitoring Network (22-day co-location). *Note*: OLS = Ordinary least squares; in the OLS regression model, MODULAIR 1 PM_2.5_ is the dependent variable, *y*, and MODULAIR 2 PM_2.5_ is the independent variable, *x*. RMSE = root-mean-square error. n = sample size.


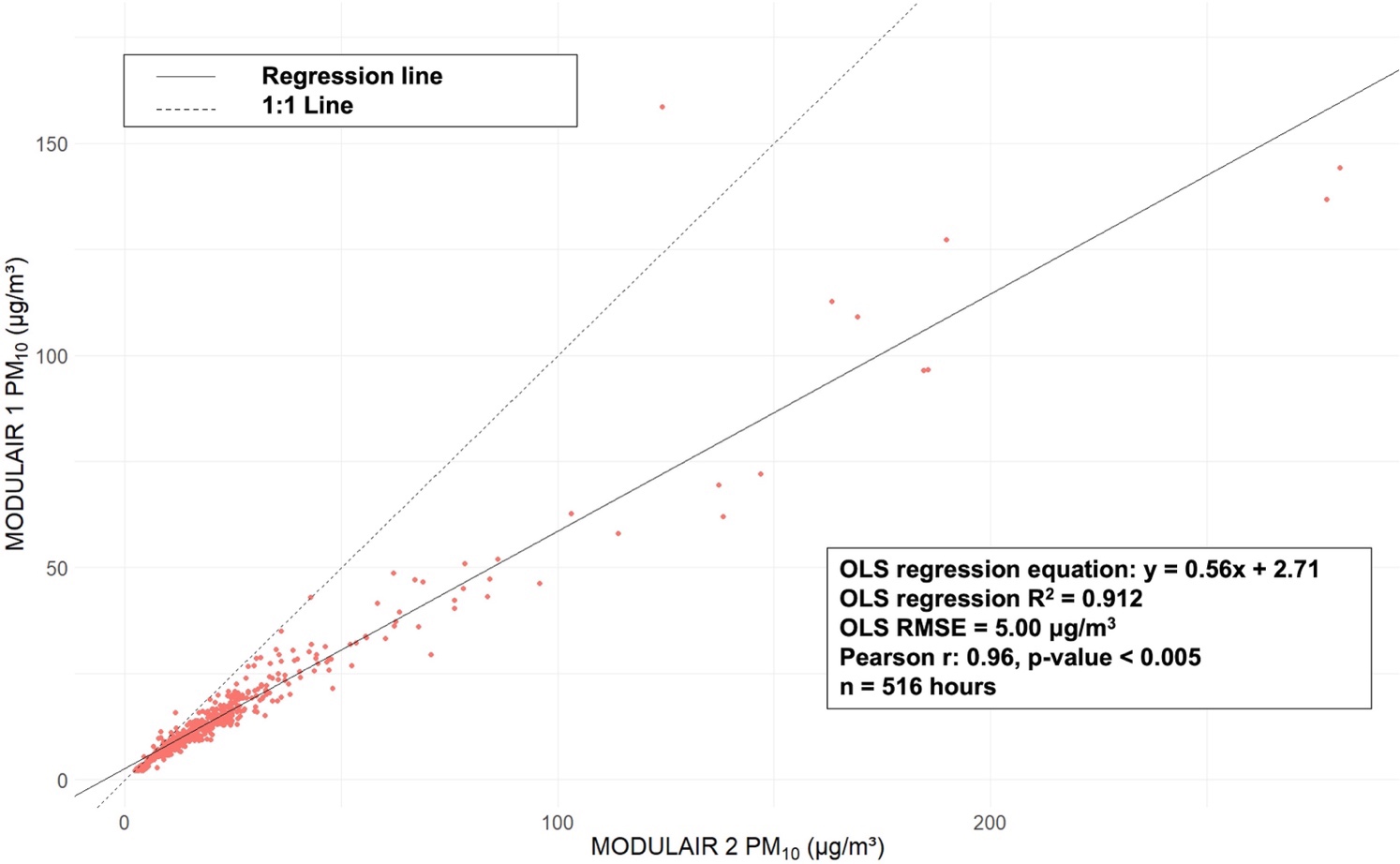


**Supplementary Figure 8.** Comparison between hourly-averaged QuantAQ MODULAIR (low-cost sensor) PM_10_ measurements (µg/m^3^) at Location 8 in the Curtis Bay Hyperlocal Air Monitoring Network (22-day co-location). *Note*: OLS = Ordinary least squares; in the OLS regression model, MODULAIR 1 PM_10_ is the dependent variable, *y*, and MODULAIR 2 PM_10_ is the independent variable, *x.* RMSE = root-mean-square error. n = sample size.


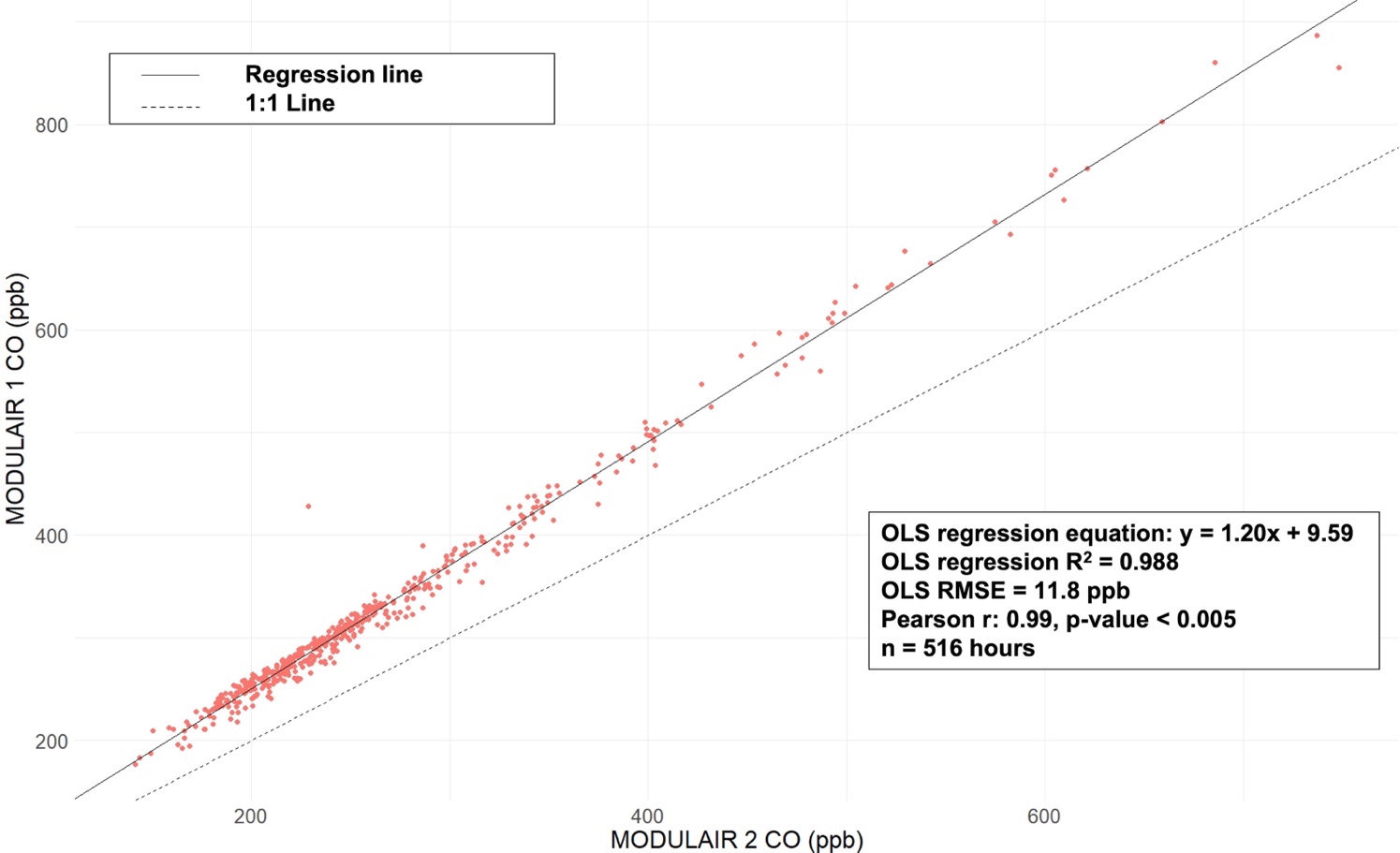


**Supplementary Figure 9.** Comparison between hourly-averaged QuantAQ MODULAIR (low-cost sensor) carbon monoxide (CO) measurements (ppb) at Location 8 in the Curtis Bay Hyperlocal Air Monitoring Network (22-day co-location). *Note*: OLS = Ordinary least squares; in the OLS regression model, MODULAIR 1 CO is the dependent variable, *y*, and MODULAIR 2 CO is the independent variable, *x.* RMSE = root-mean-square error. n = sample size.

# Supplementary References

1. Maryland Department of the Environment Ambient Air Monitoring Program. Ambient Air Monitoring Network Plan for Calendar Year 2024. Internet. Baltimore: (2023) 2023 Jan 31.

2. Maryland Department of the Environment Air and Radiation Adminstration. Lower Eastern Shore Ambient Air Quality Monitoring Project [Internet]. Maryland Department of the Environment [cited 2023 Jun 22].

3. United States Environmental Protection Agency. Criteria Air Pollutants (2022) [cited 2023 Mar 23].

4. Clements A, Duvall R, Greene D, Dye T. The Enhanced Air Sensor Guidebook. *Washington, DC: US Environmental Protection Agency* (2022).

5. Distributed Sensing Technologies. Observair Series Black Carbon Sensor – ambient Validation [Internet]. Richmond [cited 2023 Jun 22]. 1 p.].

6. Karoline Barkjohn, Andrea Clements, Amara Holder, Bob Vanderpool, Tim Hanley, Gantt B. Sensor Evaluations: The Impact of Pm2.5 Monitor Type. *National Ambient Air Monitoring Conference*; 2022 Aug 24(2022). p. 12.

7. Hagan D. Modulair Product Manual [Internet]. Somerville: QuantAQ, Inc. (2023) [updated 2023 May 3; cited 2023 Jun 20].

8. Williams R, Kilaru V, Snyder E, Kaufman A, Dye T, Rutter A, et al. Air Sensor Guidebook.
